# The ASCIZ-DYNLL1 axis promotes 53BP1-dependent non-homologous end joining and PARP inhibitor sensitivity

**DOI:** 10.1038/s41467-018-07855-x

**Published:** 2018-12-17

**Authors:** Jordan R. Becker, Raquel Cuella-Martin, Marco Barazas, Rui Liu, Catarina Oliveira, Antony W. Oliver, Kirstin Bilham, Abbey B. Holt, Andrew N. Blackford, Jörg Heierhorst, Jos Jonkers, Sven Rottenberg, J. Ross Chapman

**Affiliations:** 1 Genome Integrity Laboratory, Wellcome Centre for Human Genetics, University of Oxford, Oxford, OX3 7BN UK; 2 Division of Molecular Pathology, Oncode Institute, The Netherlands Cancer Institute, Amsterdam, 1066 CX The Netherlands; 3 St. Vincent’s Institute of Medical Research, Fitzroy, VIC 3065 Australia; 4 Genome Damage and Stability Centre, School of Life Sciences, University of Sussex, Falmer, BN1 9RQ UK; 5 MRC Brain Network Dynamics Unit, Department of Pharmacology, University of Oxford, Oxford, OX1 3TH UK; 6 Department of Oncology, Weatherall Institute of Molecular Medicine, University of Oxford, John Radcliffe Hospital, Oxford, OX3 9DS UK; 7 CRUK/MRC Oxford Institute for Radiation Oncology, University of Oxford, Oxford, OX3 7DQ UK; 8 Department of Medicine at St. Vincent’s Hospital, Melbourne Medical School, The University of Melbourne, Fitzroy, VIC 3065 Australia; 9 Institute of Animal Pathology, Vetsuisse Faculty, University of Bern, Bern, 3012 Switzerland

## Abstract

53BP1 controls a specialized non-homologous end joining (NHEJ) pathway that is essential for adaptive immunity, yet oncogenic in *BRCA1* mutant cancers. Intra-chromosomal DNA double-strand break (DSB) joining events during immunoglobulin class switch recombination (CSR) require 53BP1. However, in *BRCA1* mutant cells, 53BP1 blocks homologous recombination (HR) and promotes toxic NHEJ, resulting in genomic instability. Here, we identify the protein dimerization hub—DYNLL1—as an organizer of multimeric 53BP1 complexes. DYNLL1 binding stimulates 53BP1 oligomerization, and promotes 53BP1’s recruitment to, and interaction with, DSB-associated chromatin. Consequently, DYNLL1 regulates 53BP1-dependent NHEJ: CSR is compromised upon deletion of *Dynll1* or its transcriptional regulator *Asciz*, or by mutation of DYNLL1 binding motifs in 53BP1; furthermore, *Brca1* mutant cells and tumours are rendered resistant to poly-ADP ribose polymerase (PARP) inhibitor treatments upon deletion of *Dynll1* or *Asciz*. Thus, our results reveal a mechanism that regulates 53BP1-dependent NHEJ and the therapeutic response of *BRCA1*-deficient cancers.

## Abstract (web summary)

53BP1 is a key player in non-homologous end joining (NHEJ). Here the authors reveal an important role for the multifunctional homodimeric protein hub dynein light chain 1 (DYNLL1) in increasing the efficacy of 53BP1-mediated repair of DNA double-strand breaks (DSBs) by NHEJ.

## Introduction

To counteract the potentially carcinogenic effects of DNA damage and mutation, cells employ a complex network of DNA repair pathways^1^. DNA double-strand breaks (DSBs) are among the most toxic of genomic lesions. They arise from a variety of endogenous and exogenous sources, including ionizing radiation (IR) treatments, replication fork collapse, and as programmed intermediates of antigen receptor gene rearrangements during variable, diversity and joining (V(D)J) recombination and class-switch recombination (CSR)^2,3^. DSBs are predominantly repaired by homologous recombination (HR) and non-homologous end joining (NHEJ) pathways. HR is an essential DNA repair pathway owing to its utility in the repair of DSBs encountered during the S/G2-phases of proliferating cell populations^4^, and mutations affecting its fidelity are associated with tumourigenesis^1^. HR initiation involves the regulated nucleolytic resection of DSB termini in a 5ʹ–3ʹ direction, which is an important determinant of whether a given break is repaired by HR or NHEJ^5^. The resulting 3ʹ-tailed ends are used to invade homologous sequences on the sister chromatid, which is used as a template for accurate repair. The availability of a sister chromatid in only replicated regions of the genome ensures that HR repair is largely restricted to S and G2 phase cells, while NHEJ predominates in G1^3^. NHEJ instead involves the direct ligation of DSB ends, a mechanism utilized by developing and antigen-stimulated lymphocytes to generate programmed deletions during the diversification of antigen receptor genes. However, inappropriate activity of the NHEJ pathway is also known to promote genomic rearrangements and translocations associated with the onset of cancer. Activation of the appropriate pathway for a given DSB type, cellular context, or genomic locus is therefore crucial for the maintenance of genome stability and avoidance of deleterious errors or mutations^5^.

TP53-binding protein 1 (53BP1) is a DSB responsive protein that mediates an important and specialised branch of the NHEJ pathway^6^. 53BP1 is rapidly recruited to DSB sites, where its interaction with modified nucleosomes in DSB-flanking chromatin functions to inhibit nucleolytic DNA end resection, favouring DNA joining by NHEJ. This involves binding to a combinatorial chromatin signature consisting of methylated histone H4 (H4K20me1/2) and ubiquitinated H2A (H2AK15Ub) within DSB-associated chromatin domains^7,8^, and the recruitment of downstream effector proteins such as RIF1 (RAP1 interacting factor 1), whose binding to 53BP1 is required for efficient DNA end-protection and NHEJ^9–11^. RIF1-53BP1 complexes are essential for immunoglobulin (Ig) CSR, an NHEJ-driven deletional recombination reaction in activated B cells that mediates the replacement of excised default IgM-encoding constant (*C*) gene segments of the Ig heavy locus *(Igh)*, with downstream *C* segments of different class and function (e.g., IgG, IgE and IgA). Mice deficient in 53BP1 or RIF1 are immunodeficient owing to CSR failure^9,12–14^, defects that manifest as a result of the aberrant hyper-resection of *Igh* DSBs that generate ssDNA intermediates non-amenable to NHEJ^9,14,15^. The 53BP1 pathway also plays an equivalent but pathological role in DNA end-joining at de-protected telomeres: telomeric DNA ends exposed upon disruption of the telomere capping complex Shelterin are predominantly repaired by 53BP1-dependent NHEJ, resulting in chromosome end fusions^16^. Accordingly, uncapped telomeric DNA ends are hyper-resected in *53bp1*- and *Rif1-*deficient cells, resulting in a near complete suppression of telomere end-joining^11,17^.

53BP1-dependent NHEJ is also problematic in *BRCA1-*deficient cancers, where it mediates chromosomal rearrangements that drive oncogenesis^18,19^. In mice, germline *53bp1*-deletion suppresses the embryonic lethality and mammary tumourigenesis associated with homozygous *Brca1* loss-of-function mutations, a rescue explained by reactivation of HR^19^. Conversely, 53BP1 pathway-associated DSB repair activities underlie the synthetic lethal effect of poly-ADP ribose polymerase (PARP) inhibitor (PARPi) treatments in *BRCA1* mutation-associated cancers: genetic ablation of 53BP1 pathway components results in PARPi resistance in cellular and tumour models of *BRCA1*-deficiency^9,20,21^.

Aside from DSB repair, distinct 53BP1 protein complexes regulate the activation of p53-dependent senescence programs triggered in response to p53-activating drug treatments and prolonged mitotic transit^22–25^. Here, 53BP1’s C-terminal BRCT domains bridge p53’s interaction with the deubiquitinating enzyme USP28, catalysing de-ubiquitination events that stimulate p53 binding to, and activation of, target gene promoters^22^.

While mechanistically distinct, both NHEJ and p53-regulatory functions require 53BP1 multimerization^22,26,27^, a function thought to rely entirely on its oligomerization domain (OD; encoded within amino acid residues (a.a.) 1231–1277)^28^. NHEJ-deficits have been attributed to an inability to recruit 53BP1 to DSB-associated chromatin, as OD mutation/deletion blocks the retention of 53BP1 protein fragments at DNA damage sites, and also compromises their interaction with modified nucleosome core particles in vitro^8,28,29^. Perhaps unexpectedly, we and others recently reported that the recruitment of a full-length 53BP1 protein into IR induced foci (IRIF) was only modestly impacted by OD- deletion or mutation^22,27^. This prompted us to explore the molecular basis and function of OD-independent 53BP1 recruitment.

Here, we reveal that 53BP1 can interact with DNA damage sites independently of its OD, and this recruitment depends on interaction with the multifunctional homodimeric protein hub dynein light chain 1 (DYNLL1). We demonstrate that DYNLL1 binding promotes ordered 53BP1 oligomerization and is essential for efficient CSR and adaptive immunity in mice, while also contributing to 53BP1-dependent p53 regulation. In addition, we show DYNLL1-53BP1 interplay plays an essential role in mediating toxic NHEJ events in *Brca1* mutant cancer cells: deletion of *DYNLL1* or its transcriptional regulator ATM substrate Chk2-interacting Zn^2+^ finger protein (ASCIZ; also known as ATMIN/ZNF822) is strongly selected for in *BRCA1-*deficient tumour cells, and results in PARPi resistance in vitro and in vivo. Thus, our findings identify an important mechanism in which DYNLL1 directly regulates the fidelity of 53BP1-dependent NHEJ.

## Results

### DYNLL1 is essential for OD-independent 53BP1 recruitment

**Fig. 1:**
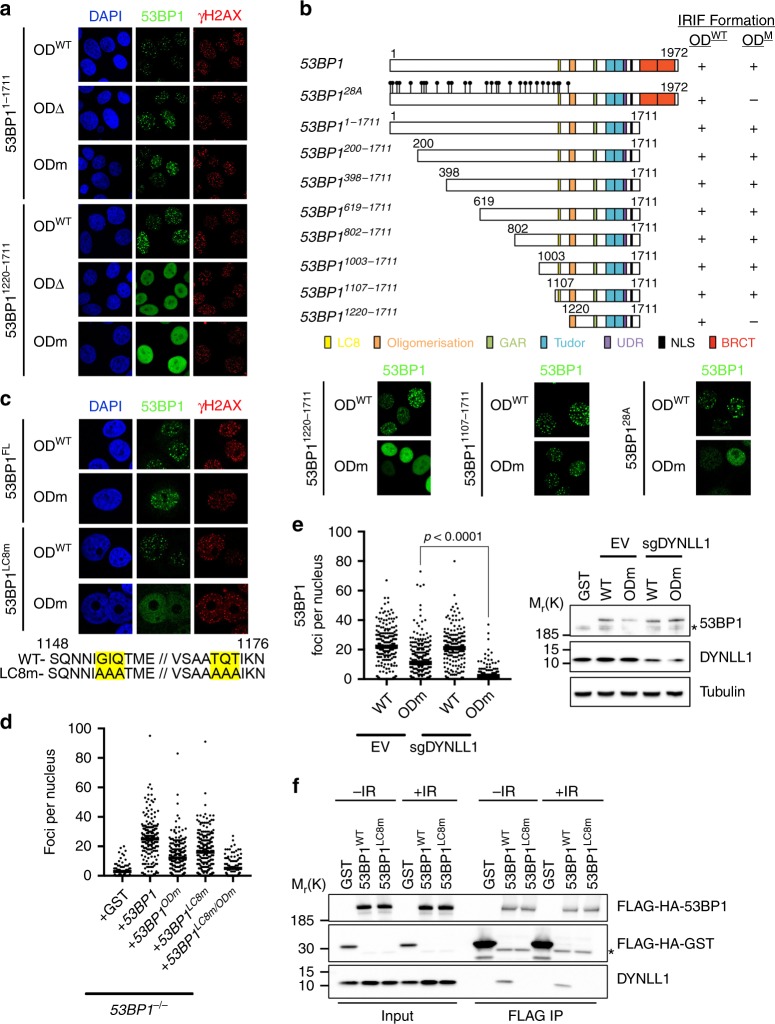
DYNLL1 promotes OD-independent 53BP1 recruitment to DSB sites. **a** *53BP1*^*-/-*^ MCF-7 cells stably expressing the indicated 53BP1 transgenes were irradiated (5 Gy), fixed 4 h later, and immunostained with anti-HA (53BP1) and anti-γH2AX antibodies. Data, representative of *n* = *3* independent experiments. ODΔ indicates deletion of amino acids 1230–1270 and ODm indicates mutation of amino acids 1258–1261 to alanine. **b** IRIF forming capability of indicated 53BP1 constructs was determined in stable cell lines as in (**a**) 4 h following 5 Gy irradiation in a minimum of *n* *=* *2* independent experiments. **c** As in (**a**-**b**), but with indicated WT and mutant 53BP1 constructs. Data, representative of *n* *=* 2 independent experiments. Amino acids 1142–1181 of 53BP1 are depicted including 6 amino acids mutated to alanine in the LC8m allele. **d** Automated 53BP1 IRIF quantification of data in (**c**). Each point represents a single nucleus with *n* *≥* 65 nuclei scored per condition. Median is indicated. Data, representative of *n* *=* 2 independent experiments. **e** Left, quantification of 53BP1 IRIF 4 h after irradiation (5 Gy). Significance determined by Mann–Whitney U Test. Right, immunoblot analysis of experimental cell lines. Each point represents a single nucleus with *n* *≥* 131 nuclei scored per condition. Median is indicated. Data, representative of *n* *=* 2 independent experiments. **f** FLAG-HA-53BP1 immuno-complexes were isolated from whole cell extracts prepared from untreated and irradiated (10 Gy, 1 h) HEK 293 T cultures, 48 h following transfection with indicated control (GST) or 53BP1 expression plasmids. Representative data, *n* *=* 2 independent experiments

The minimal domain requirements for 53BP1 DSB localization were defined in the context of a 53BP1 fragment encompassing a.a. 1220–1711^30,31^. This fragment spans 53BP1’s nuclear localization sequence (NLS), OD, tandem-tudor, and ubiquitin-dependent recruitment (UDR) domains, all of which were essential for its recruitment into IRIF^8,28^. We were therefore surprised that complete deletion of its OD domain (53BP1^ODΔ^) or Ala mutation of four key residues for OD activity (53BP1^ODm^) only negligibly impacted on 53BP1 IRIF frequencies, despite completely blocking recruitment in the context of truncated 53BP1^1220–1711^ proteins (Fig. 1a). To map the sequences in 53BP1 that mediate OD-independent recruitment, IRIF localization patterns were examined across a panel of truncated 53BP1^ODm^ proteins in which 53BP1 sequences N- and C-terminal to a.a. 1220–1711 were restored (Fig. 1b and Supplementary Fig. 1a). We found that the addition of 113 residues N-terminal to 53BP1^1220–1711^ rescued 53BP1^ODm^ recruitment into IRIF (Fig. 1b), implicating a.a. 1107–1219 in OD-independent 53BP1 recruitment.

Interestingly, alanine substitutions at 28 N-terminal [Ser/Thr]-Gln consensus ATM/ATR phosphorylation site motifs in the full-length 53BP1^ODm^ protein recapitulated the effect of a.a. 1107–1219 deletion on IRIF recruitment (Fig. 1b), and suggested that some of these Ser-Gln or Thr-Gln motifs may be involved in the OD-independent 53BP1 recruitment to DSBs. We noted that Thr-1171, one of the three Ser-Gln/Thr-Gln motifs in the region critical for OD-independent IRIF formation (a.a. 1107–1219), also serves as a binding site (consensus Thr-Gln-Thr) for the dynein light chain protein DYNLL1 (LC8)^32^. As DYNLL1 is known to function as a ubiquitous sequence-specific dimerization hub for a plethora of diverse proteins^33,34^, we considered a role for DYNLL1 in the OD-independent 53BP1 recruitment mechanism. 53BP1 a.a. 1107–1219 also contains a second DYNLL1-binding site^32^, and indeed, alanine substitution of the three anchor residues (GIQ and TQT, respectively) in each of the two DYNLL1-binding site motifs of 53BP1 (53BP1^LC8m^) completely blocked OD-independent 53BP1 IRIF formation (Fig. 1c–d). Moreover, we found that DYNLL1 colocalized with 53BP1 in nuclear foci and that this interaction was abolished by the 53BP1^LC8m^ mutations (Supplementary Fig. 1b). However, the 53BP1^LC8m^ mutation did not interfere with OD-mediated oligomerization in vitro (Supplementary Fig. 2a), or downstream RIF1 recruitment (Supplementary Fig. 2b, c). Thus, collectively, these data indicate that direct binding of DYNLL1 to the LC8-binding motifs is critical for the OD-independent recruitment of 53BP1 to DSB sites.

To further explore DYNLL1’s role in modulating 53BP1 functions, we used CRISPR-Cas9 to mutagenize the *DYNLL1* gene in *53BP1*^*-/-*^ MCF-7 cell populations stably complemented with wild type (WT) 53BP1 or 53BP1^ODm^. DYNLL1*-*depletion in these populations (without selection for stable *DYNLL1* knockout clones) caused a near complete block in 53BP1^ODm^ foci formation (Fig. 1e and Supplementary Fig. 2d, *p* *<* 0.0001), and residual 53BP1 IRIF frequencies in these experiments correlated to cells of increased DYNLL1 nuclear staining intensity (Supplementary Fig. 2e). In contrast, DYNLL1-depletion did not affect the frequencies of WT 53BP1 IRIF when the OD was intact (Fig. 1e; *p* *=* 0.1363). These experiments therefore suggested that DYNLL1-dependent binding to the LC8 motifs in 53BP1 promotes 53BP1 oligomerization, explaining the mechanism of OD-independent 53BP1 recruitment to IRIFs.

### DYNLL1-53BP1 interactions are DNA damage- and cell cycle-independent

As indicated above, one of the two LC8 motifs (LC8 motif 2) in 53BP1 also comprises an ATM/ATR phosphorylation site (Thr-1171, consensus Thr-Gln) that is phosphorylated following IR-treatment^35^, and a second putative ATM phosphorylation site (Ser-1148) is positioned only 5 a.a. residues upstream of LC8 motif 1. Thus, we considered that 53BP1-DYNLL1 interactions might be DNA damage regulated. To test this, Flag-HA tagged WT 53BP1 and 53BP1^LC8m^ mutant protein complexes were immunoprecipitated from lysates prepared from irradiated and untreated stable cell lines and analyzed for the presence of DYNLL1. In confirmation of a role for the 53BP1 LC8 motifs in mediating DYNLL1 interactions, DYNLL1 co-precipitated with wild type, but not LC8-motif mutant 53BP1 complexes (Fig. 1f). Notably, DYNLL1-53BP1 interactions were not significantly altered upon IR-treatments (Fig. 1f), discounting a role for Thr-1171 phosphorylation in regulating DYNLL1-53BP1 interactions. In addition, mutation of Ser-1148, which would be expected to form the N-terminal residue of the fully extended ~8-residue beta-strand interacting with the DYNLL1-binding groove based on known structures for DYNLL1-target complexes^36^, by itself had only a modest effect on DYNLL1 binding. Mutation of Thr-1171, however, reduced DYNLL1-binding to a greater extent, which was further reduced in concert with the Ser-1148-Ala mutation, albeit not to the same extent as the 53BP1^LC8m^ interaction-blocking mutation in which the entire 3-residue anchor motifs are disabled (Supplementary Fig. 1c). Moreover, combined alanine substitutions at Thr-1171 and Ser-1148 resulted in the near total loss of OD-independent 53BP1 IRIF (Supplementary Fig. 1d). The most likely explanation for these results is that mutation of these two phosphorylation site motifs impacts the interaction with DYNLL1 by weakening its binding sites in 53BP1 rather than by impairing their phosphorylation state, reconciling the IRIF recruitment defect of the 53BP1^ODm,28A^ mutant protein (Fig. 1b).

Next, we examined whether DYNLL1-53BP1 interactions might be regulated by cell cycle position. Cell synchronisation experiments confirmed DYNLL1 did not exhibit cell-cycle-dependent fluctuations in expression during interphase (Supplementary Fig. 3a), or mitosis (Supplementary Fig. 3b). To determine whether 53BP1 might be regulated by cell-cycle dependent modification of DYNLL1, or otherwise, 53BP1 IRIF were quantified in G1, S and G2 cell-cycle stage classified populations in WT and mutant complemented *53BP1*^*-/-*^ MCF-7 stable cell lines (Supplementary Fig. 3c). Here, the 53BP1^LC8m^ mutation did not significantly impair foci formation at any cell cycle phase (Supplementary Fig. 3d). Likewise, 53BP1^ODm^ IRIF frequencies, that rely entirely on DYNLL1-interactions (see Fig. 1c–f), were equally reduced at all cell cycle phases (Supplementary Fig. 3d). Collectively, these experiments indicate DYNLL1-53BP1 interactions are likely to be constitutive, and suggested that DYNLL1 might represent an integral component of 53BP1 complexes.

### 53BP1-DYNLL1 interactions are required for class-switch recombination

**Fig. 2:**
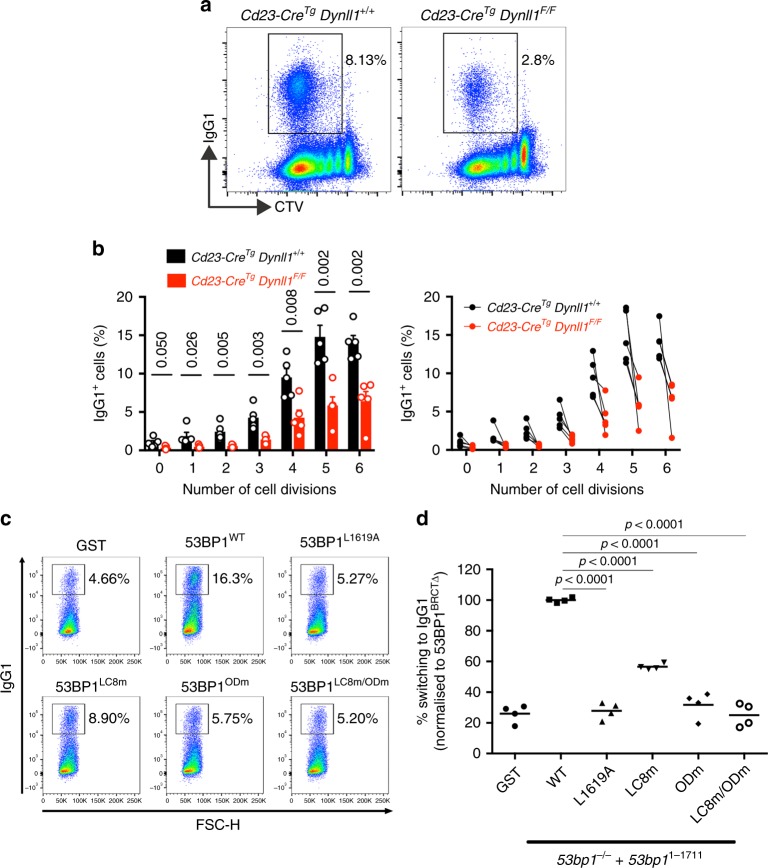
53BP1-dependent class-switch recombination requires DYNLL1. **a** In vitro CSR to IgG1 in *Dynll1*^*F/F*^ *Cd23-Cre*^*Tg*^ and control *Cd23-Cre*^*Tg*^ mature B splenocytes, 96 h following stimulation with LPS and IL-4. Representative flow cytometric plots depict IgG1 positive fraction of cells, in cells co-stained with CellTrace Violet (CTV). **b** IgG1-positive (IgG1^+^) B cells as a proportion of total B cells (%) for each cell generation as determined by CTV staining and proliferation-associated dye dilution. Significance was determined by unpaired two-tailed student’s t-test with Holm-Sidak correction for multiple comparisons. Data, *n* *=* 5 mice, mean ± SEM. **c** In vitro CSR to IgG1 in mature B splenocytes from *53bp1*^*-/-*^ mice, 96 h following stimulation with LPS and IL-4, and 72 h following retroviral complementation as indicated. Representative plots. **d** Quantification of (C). Bars represent mean, *n* *=* 4 mice. Statistical analysis by unpaired two-tailed student’s t-test

We next examined the function of DYNLL1 and DYNLL1-53BP1 interactions during CSR, which relies on 53BP1-mediated NHEJ^12,13^. DYNLL1 is essential for normal B cell development, and its deletion in the early B cell lineage using *Mb1-Cre* leads to dramatic losses in circulating and mature splenic B cell populations (Supplementary Fig. 4a)^37^. We therefore used transgenic *Cd23-Cre* driver (*Cd23-Cre*^*Tg*^*)* to delete *Dynll1* in mature B lymphocytes in *Dynll1*^*F/F*^ mice, which supported the development of normal frequencies of mature splenic B cells in which DYNLL1 protein was efficiently depleted (Supplementary Fig. 4b, c). Cultured *Dynll1*-deleted B splenocytes upon stimulation with IL-4 and LPS showed a reduction in class-switching to IgG1, and IgG1 switching frequencies in *Cd23-Cre*^*Tg*^ *Dynll1*^*F/F*^ cells were consistently reduced by >50% relative to *Cd23-Cre*^*Tg*^ controls in all cell populations that had undergone equivalent numbers of cell divisions (Fig. 2a, b). This was independently confirmed by *Cd23-Cre*-mediated deletion of the *Asciz* gene in mouse mature B cells, which encodes DYNLL1’s transcriptional regulator ASCIZ (ATMIN)^38,39^ and resulted in greatly reduced DYNLL1 expression and an equivalent reduction in class-switching efficiency (Supplementary Fig. 4d, e), consistent with previous results in B cells from *Cd19-Cre Asciz-*mutant mice^40^. The fact that CSR defects in *Dynll1-* and *Asciz-*deleted B cells could not be explained by the defective expression of *Igh* switch region germ-line transcripts (Supplementary Fig. 4f), nor the magnitude of proliferation defects in *Dynll1-*deleted B cells (Fig. 2b), was consistent with a role for DYNLL1 and its regulator ASCIZ in the end joining phase of CSR.

To exclude the possibility that indirect, 53BP1-independent, consequences of DYNLL1-deletion could account for CSR defects in *Dynll1-*deficient B cells, we next monitored the effect of LC8 binding site mutations on 53BP1-dependent CSR. LPS/IL-4-induced class-switching to IgG1 was analysed in stimulated *53bp1*^*-/-*^ primary B splenocytes upon reconstitution with retroviruses that express wild type 53BP1, or the 53BP1^ODm^, 53BP1^LC8m^, and 53BP1^LC8m/ODm^ mutant proteins, or as a negative control, the NHEJ-defective 53BP1^L1619A^ UDR mutant^8^ (Fig. 2c; note that to avoid the inefficient packaging of large *53BP1* inserts in retroviral particles, all rescue constructs express a truncated 53BP1 protein (a.a. 1–1711) that supports WT CSR frequencies^26^). As expected, 53BP1^1–1711^ expression rescued class switching in *53bp1*^*-/-*^ B cell cultures, while the repair-deficient 53BP1^L1619A^ mutant could not (Fig. 2c, d). In contrast, 53BP1^LC8m^-reconstitution only restored CSR to ~50% of WT (Fig. 2c, d), a defect consistent in magnitude to *Dynll1-*deficient B cells, confirming DYNLL1 mediates CSR via its regulation of 53BP1 complexes and their NHEJ activities. As expected^26^, *53bp1*^*-/-*^ B cells complemented with a mutant OD-domain allele (53BP1^ODm^) failed to restore CSR to any significant degree (Fig. 2c, d). Thus, while DYNLL1 can mediate OD-independent 53BP1 DSB recruitment, OD-independent recruitment alone is unable to support NHEJ during CSR, confirming a cooperation between OD- and DYNLL1-dependent oligomerization in the assembly of NHEJ-competent 53BP1 oligomers.

### DYNLL1 stimulates 53BP1 oligomerization to promote optimal chromatin interactions

**Fig. 3:**
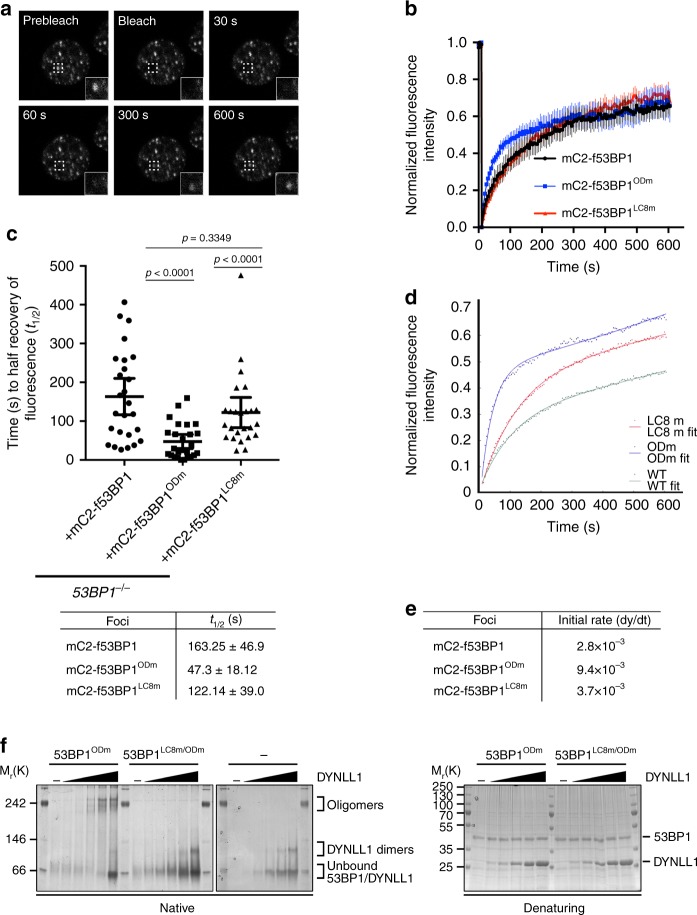
Stable 53BP1-chromatin interactions require DYNLL1-dependent and -independent oligomerization modes. **a** Representative FRAP series. Example shows recovery of WT mC2-f53BP1. Bottom right corner shows magnified region of bleached area. All FRAP experiments were performed in *53BP1*^*-/-*^ MCF-7 cells stably expressing the indicated mC2-f53BP1 fragments. **b** Mean recovery from a single FRAP experiment in cells expressing mC2-f53BP1 (*n* *=* 8), mC2-f53BP1^LC8m^ (*n* *=* 9), or mC2-f53BP1^ODm^ (*n* *=* 10), mean ± SEM. Data, representative of *n* *=* 3 independent experiments. **c** Time for half-recovery of maximum final fluorescence for IRIF in individual cell expressing mClover2-WT (*n* *=* 26), mC2-f53BP1^LC8m^ (*n* *=* 26), or mC2-f53BP1^ODm^ (*n* *=* 25) proteins. Mean ± CI (95%). *P-*values, Mann–Whitney U test. **d** Curves of best fit for three aggregated experiments. Data points represent the mean of *n* ≤ 25 determinations. **e** Calculated initial rates (dy/dt) for the curves generated in (**d**). **f** Purified Smt3-His_6_-tagged DYNLL1 was incubated with Smt3-His_6_-tagged 53BP1^ODm^ or 53BP1^LC8m/ODm^ fragments (a.a. 1131–1292) as indicated. 1 μg of 53BP1 was added to each reaction with increasing concentrations of DYNLL1. Molar ratios of DYNLL1:53BP1 from left to right; 1:4, 1:2, 1:1, 2:1, 4:1. Samples were fractionated by native (8%) or denaturing (12%) PAGE and stained with Coomassie Brilliant Blue. Data, representative of *n* *=* 2 independent experiments

To ascertain why DYNLL1-mediated 53BP1 recruitment alone is insufficient for DNA repair, we investigated DYNLL1’s contribution to 53BP1-chromatin interaction dynamics at DSB sites. Stable *53BP1*^*-/-*^ MCF-7 cell lines were established that expressed equivalent levels of an mC2 (mClover2)-tagged 53BP1 a.a. 1107–1711 fragment, comprising WT (mC2-f53BP1) or mutated versions of the OD (mC2-f53BP1^ODm^) or LC8 motifs (mC2-f53BP1^LC8m^). The mobility of each protein in IRIF was then calculated in fluorescent recovery after photobleaching (FRAP) experiments (Fig. 3a). Fluorescence recovery of mC2-f53BP1^ODm^ was >3-fold faster than that of WT mC2-f53BP1 protein, as calculated from the half-time for recovery to maximum fluorescence: *t*_*1/2*_ *=* 47.3 s ± 18.12 and 163.25 s ± 46.9, for mC2-f53BP1^ODm^ and mC2-f53BP1 proteins, respectively (Fig. 3b, c). Thus, DYNLL1-mediated 53BP1 oligomerization alone is insufficient for stable chromatin interaction at DSB sites. These results confirm canonical DYNLL1-independent oligomerization is primarily responsible for 53BP1 interactions with DSB-associated chromatin. To control for variation in fluorescence recovery endpoints between experimental repeats (Fig. 3d), we analyzed the rate of fluorescence recovery in a manner that, unlike *t*_*1/2*_, was not related to the endpoint. Thus, we computationally modelled the fluorescence recovery data of three independent experiments (each comprising *n* > 8 cells per genotype) to calculate the initial rate of fluorescence recovery immediately after photobleaching (Fig. 3d). Initial rate calculations provide a concentration-independent measurement of fluorescence recovery that is independent of fluorescence recovery endpoint. Consistent with *t*_*1/2*_ measurements, the initial recovery rates of each protein reproduced the trends seen in Fig. 3b, c, with large, and moderate increases in the recovery rates of mC2-f53BP1^ODm^ and mC2-f53BP1^LC8m^ mutant proteins, respectively, relative to WT mC2-f53BP1 (Fig. 3e). These data confirmed an important role for DYNLL1 in stabilizing OD-dependent 53BP1-chromatin interactions.

To determine the mechanism by which DYNLL1 contributes to 53BP1 function, we next probed DYNLL1’s ability to promote 53BP1 oligomerization in vitro. Thus, purified DYNLL1 was titrated into binding reactions that contained a recombinant monomeric 53BP1 protein fragment encoding the two LC8-binding motifs and a mutated OD domain (a.a. 1131–1292). Reactions were then resolved by native PAGE to ascertain DYNLL1’s ability to stimulate OD-independent 53BP1 oligomerization. In these experiments, increasing DYNLL1 to levels equimolar and above that of the 53BP1 fragment, stimulated its robust oligomerization as determined by a marked increase in higher molecular weight protein complexes (Fig. 3f). In confirmation of a role for the 53BP1 LC8 binding motifs in mediating the assembly of DYNLL1-bridged 53BP1 oligomers, higher molecular weight complexes did not form when both LC8-binding motifs in the 53BP1 fragment were mutated, and these reactions were characterised by an increased presence of ligand-free DYNLL1 dimers (Fig. 3f). Taken together, our data indicate that optimal 53BP1 oligomerization is mediated via a cooperation between its OD-domain (Fig. 1a, b), and the binding of DYNLL1 to its upstream binding sites in 53BP1 (Fig. 3f). We therefore conclude that the intermolecular affinity afforded by DYNLL1-53BP1 interactions is sufficient to promote 53BP1 oligomerization, yet in isolation is insufficient to efficiently tether 53BP1 complexes to DSB-associated chromatin. We instead propose that DYNLL1 may provide order to large oligomeric 53BP1 complexes, a function required for optimal chromatin interactions and its associated DNA repair function.

### PARPi hypersensitivity in *Brca1-*deficient cells requires 53BP1-DYNLL1 cooperation

**Fig. 4:**
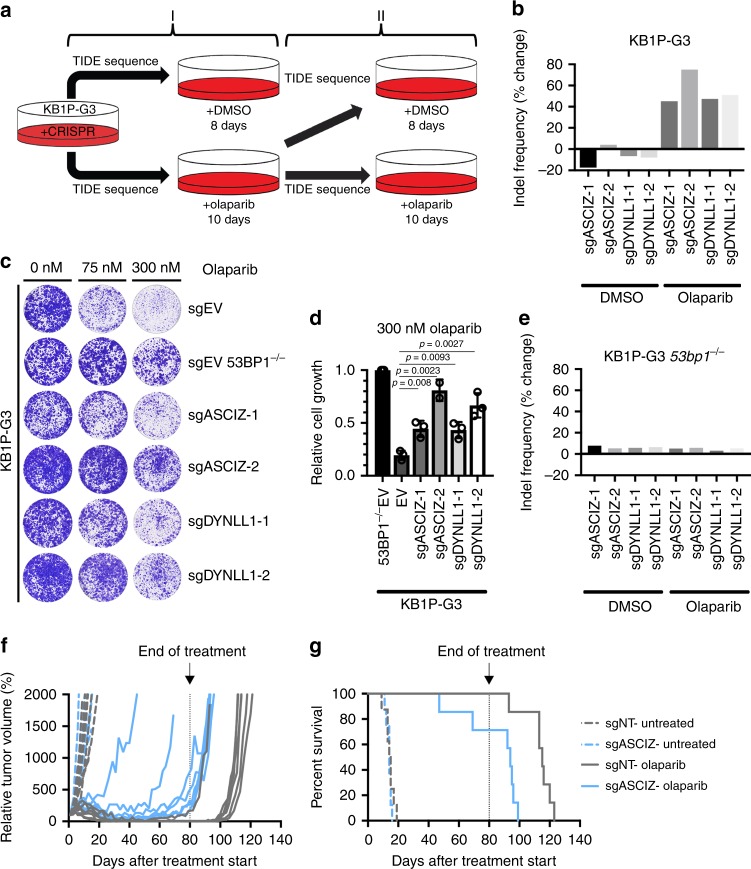
ASCIZ and DYNLL1 co-regulate 53BP1-dependent olaparib sensitivity in *Brca1-*deficient cells. **a** Schematic of experiments to determine olaparib resistance. **b** Change in the percent of edited alleles for the indicated target genes in KB1P-G3 cells after growth in DMSO or olaparib (75 nM). Data, representative of *n* *=* 3 independent experiments. **c** Representative images from the second round of olaparib growth (II) in either DMSO or olaparib. **d** Quantification of growth in olaparib (300 nM) after transduction with indicated guide RNAs. Quantification of *n* *=* 3 independent experiments, each with three technical replicates. Mean ± SD. Significance determined by unpaired two-tailed t test. **e** Change in the percent of edited alleles in *53bp1*^*-/-*^ *p53*^*-/-*^ KB1P-G3 cells after growth in DMSO or olaparib (75 nM). Data, representative of *n* *=* 2 independent experiments. **f** Relative tumour volume of transplanted organoids after treatment with olaparib. *n* *>* 7 animals per condition. **g** Kaplan–Meier plot indicating survival of organoid-transplanted animals. Significance between sgNT—olaparib and sgASCIZ—olaparib was determined by Log-rank Mantel–Cox test (*p* *=* 0.0018). *n* *>* 7 animals per condition

The fact that DYNLL1-mediated regulation of 53BP1 oligomerization enhances 53BP1-dependent CSR next prompted us to test its importance more broadly in 53BP1-dependent NHEJ. The efficacy of PARPi in killing BRCA1*-*deficient cells is dependent on 53BP1, and its loss in BRCA1*-*deficient cell-lines and mice results in resistance to PARPi-induced cell death^9,19–21^. We therefore theorized that reduced efficiency of 53BP1-dependent NHEJ upon DYNLL1 depletion might likewise confer PARPi resistance to BRCA1-deficient cells. Using CRISPR-Cas9, we mutagenized the *Dynll1* and *Asciz* genes in the KB1P-G3 *Brca1*^*-/-*^ *p53*^*-/-*^ murine mammary tumour cell line^20^, and monitored locus-specific indels within cell populations by deconvolution of complex Sanger sequencing traces derived from Cas9 cleavage site-spanning PCR amplicons using the Tracking of Indels by Decomposition (TIDE) algorithm^41^ (Supplementary Fig. 5a). To determine whether mutation of *Dynll1* or *Asciz* provided a selective advantage in KB1P-G3 cells due to PARPi resistance, the change in percentage of edited alleles between the starting population and the surviving fraction was compared, before, and after, outgrowth in the presence or absence of the PARPi olaparib (Fig. 4a and Supplementary Fig. 5a). In all experiments in which the initial indel frequency at the *Dynll1* and *Asciz* loci was below saturation (<85%) in treatment naive cells, olaparib treatments selected for striking increases in indel frequency (Fig. 4b and Supplementary Fig. 5b). These effects were coupled to reductions in DYNLL1 protein levels when compared to untreated controls (Supplementary Fig. 5c), collectively confirming that both proteins are required for the hypersensitivity of *Brca1-*deficient cells to PARPi. Cas9-dependent mutagenesis of either gene in KB1P-G3 cells also conferred olaparib resistance in clonogenic survival experiments (Fig. 4c, d). Importantly, the Olaparib resistance in *Asciz*-mutated KB1P-G3 cells could be completely reversed by transgene-mediated re-expression of exogenous DYNLL1 (Supplementary Fig. 6a–c), confirming the importance of ASCIZ-mediated DYNLL1 transcription in the response of BRCA1-deficient cells to PARPi. However, neither selection for CRISPR-Cas9-dependent *Dynll1* or *Asciz* locus editing, nor reduced DYNLL1 expression, was observed when equivalent experiments performed in *53bp1*^*-/-*^ KB1P-G3 cells (Fig. 4e and Supplementary Fig. 5d, e), indicating that DYNLL1’s role in mediating the PARPi sensitivity of *Brca1*-deficient cells occurs predominantly via its regulation of 53BP1 activities.

We next examined whether the ASCIZ-DYNLL1 axis was required for PARPi sensitivity in vivo. ASCIZ and DYNLL1 have previously been shown to have equivalent effects on the development and expansion of *Myc*-driven cancers^37,42^, thus we monitored the growth of Cas9-expressing *Brca1*^*-/-*^ *p53*^*-/-*^ transplanted mouse cancer organoids^43^ transduced with control or *Asciz*-targeting gRNA (Supplementary Fig. 5f, g). Olaparib treatment significantly delayed the onset of tumour growth in transplanted animals relative to an untreated cohort. However, PARPi-dependent inhibition of tumour growth was attenuated in *Asciz*-edited tumours (Fig. 4f), and resulted in decreased overall survival relative to control olaparib-treated cohorts (Fig. 4g). These data demonstrated that regulation of 53BP1 by the ASCIZ-DYNLL1 axis supports the efficacy of olaparib treatments in selectively killing *Brca1-*deficient cancer cells, confirming the importance of DYNLL1-53BP1 interactions in regulating 53BP1-dependent DNA repair.

### DYNLL1-binding sites contribute to 53BP1-dependent Nutlin-3 sensitivity

**Fig. 5:**
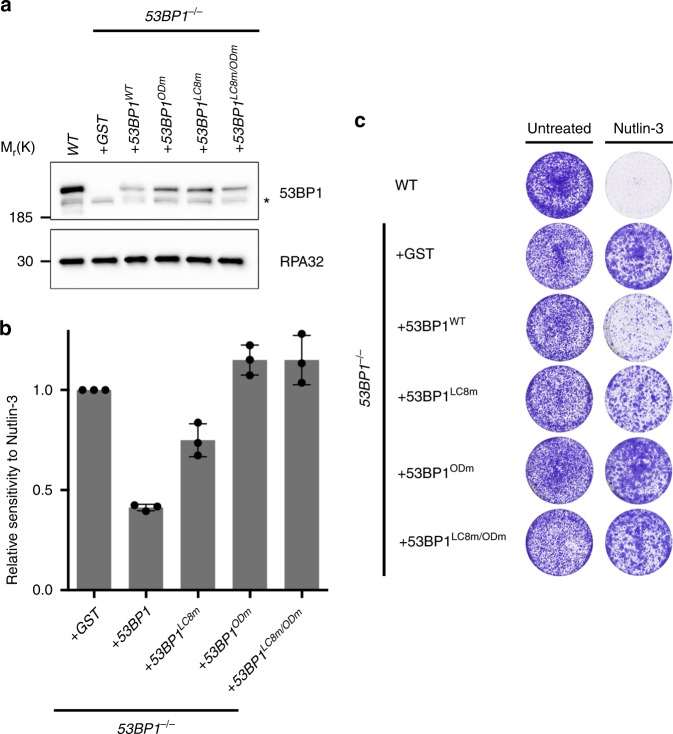
DYNLL1 is required for 53BP1-dependent p53 responses to Nutlin-3. **a** Immunoblot analysis of the MCF-7 cell lines used in (**a**) with anti-53BP1 or anti-RPA32 antibodies prior to N3 treatment. **b** Quantification of *n* *=* 3 independent experiments represented in (**a**), each performed in triplicate. Mean ± SD. **c** Indicated parental or stably complemented *53BP1*^*-/-*^ MCF-7 cell lines were incubated in the presence (11 days) or absence (7 days) of Nutlin-3 (4 µM)

In addition to its role in the DNA damage response, 53BP1 also contributes to p53-dependent transcriptional senescence programs and the corresponding cellular sensitivity to the treatments with MDM2 inhibitor Nutlin-3^22,44^. Interestingly, the 53BP1^LC8m^ mutant, in contrast to wild type 53BP1^22^, was compromised in their ability to restore the sensitivity of *53BP1*^*-/-*^ MCF-7 to Nutlin-3 (Fig. 5a–c). This agrees with a model in which DYNLL1 is likely to enforce optimal 53BP1 oligomerization, and is consistent with our published findings in which efficient 53BP1 oligomerization was similarly essential for 53BP1-dependent regulation of p53^22^.

## Discussion

**Fig. 6:**
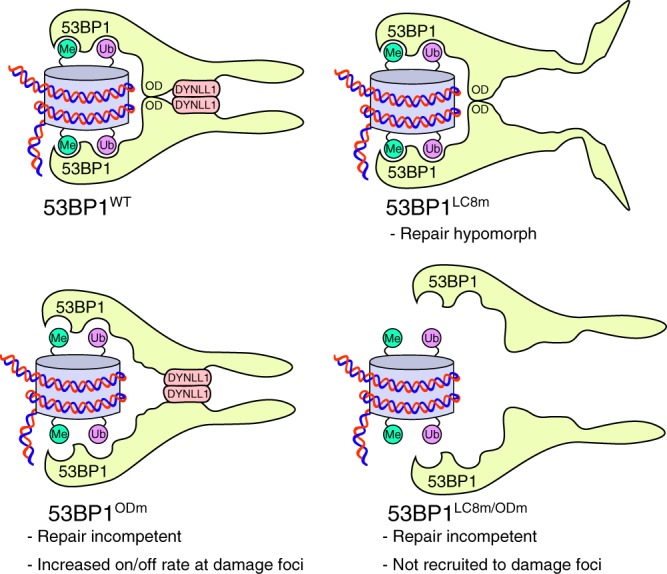
DYNLL1 and the OD mediate a bivalent mode of 53BP1 oligomerization. 53BP1 localisation and function is dependent upon bivalent OD- and DYNLL1-mediated modes of oligomerization (top, left). OD-mediated oligomerization alone is sufficient for chromatin binding but results in severe DSB repair defects (top, right). DYNLL1-mediated oligomerization is also sufficient to support recruitment of 53BP1 to damage sites, but does not support DNA repair (bottom, left). Loss of both OD and DYNLL1-mediated modes of oligomerization eliminates 53BP1 recruitment to DNA damage sites and all associated repair functions (bottom, right)

Taken together, our findings reveal DYNLL1 to be an integral component of oligomeric 53BP1 complexes through which it plays an important role in 53BP1-dependent NHEJ. We speculate that DYNLL1-binding to 53BP1 may provide order to 53BP1 oligomers within chromatin-binding domains, enhancing productive chromatin interactions that enhance effective resection inhibition and enable for efficient NHEJ. Deficiencies in either DYNLL1 or its binding sites in 53BP1, lead to substantial CSR defects. Given that 53BP1 is similarly important for resection inhibition and NHEJ during V(D)J recombination, a loss of which manifests in the defective development of B and T lymphocyte lineages^45–47^, it is plausible that 53BP1-associated V(D)J recombination defects that accompany loss of DYNLL1-53BP1 interactions might contribute to the B cell lineage development defects recently reported in *Dynll1*-deficient mice^37^. We also found that DYNLL1 contributes to DNA damage-independent 53BP1 functions in the response to the MDM2 inhibitor Nutlin-3 (Fig. 5a–c). This agrees with a model in which DYNLL1 enforces optimal 53BP1 oligomerization, and is consistent with our published findings in which efficient 53BP1 oligomerization was similarly essential for 53BP1-dependent regulation of p53^22^. Our results therefore suggest bivalent oligomerization modes are necessary to coordinate the assembly of 53BP1 molecules into ordered, functionally competent multimeric complexes, insights that will be relevant for understanding other macromolecular complexes organized by DYNLL1 (Fig. 6). In identifying DYNLL1 and ASCIZ as essential mediators of 53BP1-dependent PARPi sensitivity in *Brca1-*deficient cancer cells, our findings provide a molecular explanation for the identification of both proteins as PARPi resistance factors in *BRCA1-*deficient cells in recently published genetic screens^48,49^, and reveal a previously unanticipated mechanism in which 53BP1 misregulation could lead to PARPi resistance in the clinic.

## Methods

### Cell lines and culture conditions

Cell lines used in this study are MCF-7 female human breast adenocarcinoma, RPE-1 female human retinal pigment epithelial, human female embryonic kidney (HEK) 293T cells (including the BOSC23 derivative cell line), and KB1P-G3 *Brca1*^*-/-*^ *p53*^*-/-*^ cells which were isolated from a female mouse mammary tumour. All cell lines were checked for mycoplasma contamination. KB1P-G3 and the KB1P-G3 *53bp1*^*-/-*^ derivative were both kindly provided by Sven Rottenberg (NKI, Netherlands and University of Bern). All cell lines were cultured in DMEM—high glucose (D6546, Sigma-Aldrich) supplemented with 10% FBS, Pen-Strep, and 2 mM L-glutamine. Cultures were grown at 37 °C with 5% CO_2_. KB1-P3 (G3) and all derivative cells lines were additionally grown in 3% oxygen. Primary B cells were isolated from red blood cell-lysed single-cell suspensions of *53bp1*^*-/-*^ C57BL/6J mouse spleens and cultured in RPMI supplemented with 10% FCS, 100 U/ml penicillin, 100 ng/ml streptomycin, 2 mM L-glutamine, 1x MEM nonessential amino acids, 1 mM sodium pyruvate and 50 μM β-mercaptoethanol.

### Mouse models

Mice harbouring conditional *Dynll1* or *Asciz* alleles (MGI ID: 5755433 and 5463697, respectively) were intercrossed with *Cd23-Cre* transgenic mice (MGI ID: 3803652) to generate *Cd23-Cre*^*Tg*^ *Dynll1*^*F/F*^ and *Cd23-Cre*^*Tg*^ *Asciz*^*F/F*^ experimental and *Cd23-Cre*^*Tg*^ control animals. Mouse breeding and tissue collection was performed according to the Australian Code for the Care and Use of Animals for Scientific Purposes, 8th Edition (2013), and approved by the St. Vincent’s Hospital Melbourne Animal Ethics Committee, approval number 002/17. *53bp1*^*-/-*^ C57BL/6J mouse breeding and tissue collection was carried out according to the Animals (Scientific Procedures) Act (ASPA) under a Home Office project license to JRC.

### Gene editing and complementation

CRISPR-Cas9 editing was carried out with gene-specific gRNAs integrated into a version of lentiCRISPR v2 (Addgene #52961) modified for blasticidin (lentiCRISPR-Bsr) resistance. Lentivirus was generated by co-transfection of lentiCRISPR-Bsr, pHDM-tat1b, pHDM-G, pRC/CMV-rev1b, and pHDM-Hgpm2 into HEK 293T cells using 1.29 μg polyethylenimine per 1 μg DNA in Opti-MEM (Thermo Fisher, 31985062). Viral supernatants were harvested at 48 and 72 h and passed through a 0.45-μm syringe filter. Two rounds of viral transduction were carried out on target cell populations in the presence of 4 μg/ml polybrene before selection in 10 μg/ml blasticidin. Once all cells in a non-transduced control population had died, the CRISPR population was removed from blasticidin selection. Stable transgene expression was similarly achieved by two rounds of lentiviral transduction with viral supernatants generated using the same procedure.

### Immunofluorescence

For analysis of fixed samples, cells were seeded (1.5 × 10^5^) on glass coverslips (13 mm) in six-well plates 2 days before irradiation. IRIF formation was induced with γ-irradiation (5 Gy) and cells were fixed in 2% paraformaldehyde after 4 h recovery. Fixed cells were rinsed with PBS and then permeabilized in a 0.2% Triton X-100 PBS solution. After 15 min of blocking (3% BSA, 0.1% Triton X-100 in PBS) coverslips were incubated 1 h in a humidity chamber with primary antibodies diluted in blocking solution. The following primary antibodies were utilized at the indicated concentrations: mouse anti-HA (1:200, HA.11 Biolegend), rabbit anti-γ-H2AX (1:500, Epitomics), rabbit anti-Cyclin A (1:200, Santa Cruz sc-751), rabbit anti-DYNLL1 (1:250, AbCam Ab51603). The coverslips were rinsed three times with PBS containing 0.1% TX-100 and then incubated 1 h in a humidity chamber with secondary antibody. The following secondary antibodies were utilized at the indicated concentrations: goat anti-mouse Alexa Fluor 488 (1:500, Invitrogen A-11001), goat anti-rabbit Alexa Fluor 568 (1:500, Invitrogen A-11011). Coverslips were then washed three times in PBS containing 0.1% TX-100, once in PBS, and mounted on glass microscope slides using ProLong^®^ Gold antifade reagent with DAPI (Life Technologies, P36935).

In experiments involving EdU incorporation, cultures were pulsed with 40 μM EdU for 10 min immediately preceding irradiation. After fixation, EdU was labelled using the Click-iT™ EdU Alexa Fluor 647 Imaging Kit (Thermo Fisher, C10340) according to the manufacturer’s protocol. This was done immediately prior to antibody staining as described above.

Images for the initial mapping of the LC8 domain within the 53BP1 N-terminus (Fig. 1) were acquired on a Zeiss LSM510 META confocal imaging system (Fig. 1). RIF1 IRIF (Supplementary Fig. 2b, 2c) were analyzed on a Leica SP8 SMD X confocal microscope. All other fixed IRIF images were acquired on an Olympus Epi-fluorescence MMI CellEctor widefield microscope. Quantitative analysis of IRIF number and staining intensity was performed using Cell Profiler (Broad Institute). All image visualization and processing was done using Fiji.

### Olaparib sensitivity assays

KB1P-G3 and KB1P-G3 *53bp1*^*-/-*^ cells were subjected to two rounds of lentiviral transduction with viral supernatant generated using lentiCRISPR-Bsr. After selection in blasticidin (10 μg/ml), samples were collected to isolate genomic DNA (gDNA) immediately prior to seeding for olaparib sensitivity. Blasticidin-resistant populations were seeded in six-well plates at a density of 10^4^ cells per well (5 × 10^3^ for the KB1P-G3 *53bp1*^*-/-*^ derivative) in the presence of olaparib or DMSO and grown at 37 °C (5% CO_2_ and 3% O_2_). Medium was refreshed at 4 days and 8 days. After 10 days outgrowth, cultures were expanded in fresh medium for 1 week in 6 cm dishes before harvesting gDNA. PCR fragments encompassing the CRISPR cut site were amplified from gDNA using Q5 polymerase (NEB, M0491), sequenced (GATC Biotech), and analyzed by Tracking of Indels by Decomposition (TIDE: https://tide.deskgen.com/). Surviving cells were collected and three replicates were plated in DMSO and three in olaparib for viability analysis. DMSO and olaparib-treated cells were stained with crystal violet (0.5% (w/v) crystal violet in 25% methanol) after 8 days and 10 days growth (37 °C, 5% CO_2_ and 3% O_2_), respectively. Crystal violet stained cells were dissolved in a 10% (v/v) acetic acid solution a minimum of 24 h after staining and the OD_595_ was measured as a quantitative metric of relative growth.

### Nutlin-3 sensitivity assay

Cells were seeded in triplicate at a density of 1.25 × 10^4^ per well in a six-well plate in the presence (11 days growth) or absence (7 days growth) of Nutlin-3 (4 μM, Cayman Chemicals, added 16 h after seeding). Cells were stained with crystal violet and quantified as described for olaparib sensitivity assays. These experiments were performed in triplicate.

### Protein extraction and western blotting

Protein extracts were prepared in ice cold benzonase cell lysis buffer (40 mM NaCl, 25 mM Tris pH 8.0, 0.05% SDS, 2 mM MgCl_2_, 10 U/ml benzonase, and cOmplete™ protease inhibitor cocktail (Roche, 04693159001). Briefly, cells were harvested and washed once in ice cold PBS before resuspending in lysis buffer. After 10 min incubation on ice, protein concentration was calculated by Bradford (Bio-Rad, 500-0006) assay with reference to a standard curve generated with BSA. Extracts were mixed with 3X Laemmli buffer and heated at 95 °C for 5 min.

Extracts were fractionated on pre-cast NuPAGE™ 4–12% 1.0 mm Bis-Tris polyacrylamide gels (Life Technologies, NP0322) and transferred to nitrocellulose membranes. After blocking with 5% milk and washing in PBST, membranes were incubated overnight in primary antibody. Primary antibodies used in this study are: anti-53BP1 (Novus Biological, NB100-304, 1:2500), anti-DYNLL1 (Abcam, ab51603, 1:1000), anti-α-tubulin (Sigma-Aldrich, TAT-1, 1:10000), anti-Cyclin A (Santa Cruz, sc53227, 1:1000), anti-HA (BioLegend, 501901, 1:2000), and anti-RPA32 (Abcam, ab16855, 1:1000), anti-β-actin (Sigma-Aldrich, A1978, 1:2000). Membranes were then probed with HRP-conjugated goat anti-mouse (Thermo Fisher, 62-6520) or goat anti-rabbit (Thermo Fisher, 65-6120) secondary antibodies for 1 h (1:20000). The membranes were then developed using Clarity™ Western ECL Substrate (Bio-Rad, 170-5061) and visualized on a Gel Doc™ XR System (Bio-Rad).

### Class switch recombination

B cells were purified from red blood cell-lysed single-cell suspensions of 8–16-week-old *53bp1*^*-/-*^ C57BL/6 J mouse spleens by magnetic negative selection using a B Cell Isolation Kit (Miltenyi Biotec, 130-090-862). No controls were employed for gender and randomization methods were not used to allocate animals to control and experimental groups. B cells (7.5 × 10^5^ per well in a six-well plate) were cultured in RPMI supplemented with 10% FCS, 100 U/ml penicillin, 100 ng/ml streptomycin, 2 mM L-glutamine, 1x MEM nonessential amino acids, 1 mM sodium pyruvate and 50 μM β-mercaptoethanol. B cells were stimulated with 5 μg/ml LPS (Sigma, L7770-1MG) and 10 ng/ml mouse recombinant IL-4 (Peprotech, 214-14-20). Cultures were grown at 37 °C with 5% CO_2_ under ambient oxygen conditions.

Retroviral supernatants were collected 48 h after co-transfection of BOSC23 cells with 7 μg pCL-Eco and 7 μg pMX-IRES-GFP-derived plasmids (both kindly provided by A. Nussenzweig) containing cloned transgene inserts in the presence of FuGENE 6 (Promega, E2691). Viral supernatants were passed through a 0.45 μm syringe filter. One round of viral transduction was carried out on B cells 24 h after stimulation in the presence of 2.5 μg/ml polybrene, 20 mM HEPES, 5 μg/ml LPS and 10 ng/ml mouse recombinant IL-4. B cells underwent spinoculation at 850×*g* for 90 min at 30 °C. After a rest period of 4 to 6 h, the viral supernatants were removed, and supplemented RPMI was added to the cells.

Three days after transduction, infected B cells were analysed for the percentage of GFP and IgG1 double-positive cells using a FACSCanto (Becton Dickinson); analysis was performed using FlowJo v10. Cells were resuspended in PBS with 2% BSA and 0.025% sodium azide, blocked with Mouse BD Fc Block™ (1:500, BD Pharmingen 553141), and immunostained with biotinylated rat anti-mouse IgG1 (1:100, BD Pharmingen 553441), and Streptavidin APC (1:500, eBioscience 17-4317-82). Live/dead cells were discriminated after staining with Zombie Aqua viability dye (1:200, BioLegend 423102).

For CSR analyses of *Cd23-Cre*^*Tg*^ *Dynll1*^*F/F*^ and *Cd23*-*Cre*^*Tg*^ *Asciz*^*F/F*^ mice, experiments were performed as above except that purified B cells were activated with 10 µg/ml LPS (Invivogen, tlrl-3pelps) and 1:100-diluted conditioned murine IL-4 supernatant (kind gift of Andreas Strasser, Walter and Eliza Hall Institute, Melbourne) for 4 days, and then stained with CellTrace Violet (Thermo Fisher Scientific, C34557) and rat anti-mouse IgG1-APC (BD Pharmingen, 550874).

To analyse the transcription of *Igh* germline transcripts, 3 × 10^6^ MACS-purified B cells from *Cd23*-*Cre*^*Tg*^, *Cd23*-*Cre*^*Tg*^ *Asciz*^*F/F*^, or *Cd23-Cre*^*Tg*^ *Dynll1*^*F/F*^ mice were cultured with LPS (15 µg/mL) and IL-4 (1/100) for 0 to 3 days. After treatment, cells were washed once with ice-cold PBS and cell pellets were snap frozen on dry ice and stored at −80 °C before RNA extraction. Total RNA was extracted by using ISOLATE II RNA Micro Kit (Bioline BIO-52075) following the manufacturer’s protocol. 300 ng purified RNA was subjected to reverse transcription with oligo(dT) and random primers using the SuperScript^®^ III First-Strand Synthesis System (Invitrogen™ 18080-051). qPCR reagents were mixed as follows: 4 µl cDNA (6 ng), 0.5 µl Primer mix (300 nM each), 5 µl SYBR™ Select Master Mix (Applied Biosystems™ 4472908), 0.5 µl RNAase/DNAase free water. qPCR conditions were set as follows: 1 cycle (10 min at 95 °C), 45 cycles (15 s at 95 °C), 60 s at 60 °C.

After each PCR run, a disassociation curve program was added to validate the primer specificity. The standard curve method of comparative quantification was employed to quantify the results^50^. Results for the *Iγ1-Cγ1* germline transcript were normalized to the *Cd79b* input control, and expressed as percentage of maximum expression. Primer sequences were the same as previously used^51^: Cd79b_forward (5ʹ-TGTTGGAATCTGCAAATGGA-3ʹ), Cd79b_reverse (5ʹ-TAGGCTTTGGGTGATCCTTG-3ʹ), Iγ1-Cγ1_forw (5ʹ-TCGAGAAGCCTGAGGAATGTG-3ʹ), Iγ1-Cγ1_rev (5ʹ-GGATCCAGAGTTCCAGGTCACT-3ʹ).

### Immunoprecipitation

One day before transfection, 4.5 × 10^6^ HEK 293T cells were seeded in antibiotic-free DMEM in a 10 cm plate for each condition. 24 h after plating, cultures were transfected with 10 μg 53BP1 pLV-EF1α-FLAG-HA Puro or 53BP1^LC8m^ pLV-EF1α-FLAG-HA Puro using polyethylenimine. 24 h after transfection, each culture was trypsinized and re-plated in 2 × 10 cm plates. After 24 h, one plate was X-ray irradiated (10 Gy) and both (irradiated and non-irradiated control) were harvested 1 h after irradiation. Cells were lysed in benzonase-containing lysis buffer (20 mM HEPES [pH 7.9], 40 mM KCl, 2 mM MgCl_2_, 10% glycerol, 0.5% NP40, 0.05% [v/v] phosphatase inhibitor cocktails 2 and 3 (Sigma-Aldrich), 1X protease inhibitor (Roche), and benzonase [50 U/ml]) on ice for 10 min. KCl was adjusted to a final concentration of 450 mM and rotated at 4 °C for 30 min before clarification. Clarified lysates were diluted to a final salt concentration of 150 mM KCl with No-salt equilibration buffer (20 mM HEPES [pH 7.9], 0.5 mM DTT, 0.05% [v/v] phosphatase inhibitor cocktails 2 and 3 (Sigma-Aldrich), 1X protease inhibitor (Roche)) and 2 mg of protein was incubated with anti-FLAG M2 magnetic beads for 2 h. Bead-substrate complexes were washed five times in wash buffer (20 mM HEPES [pH 7.9], 100 mM KCl, 0.5 mM DTT, 1X protease inhibitor (Roche)) at 4 °C before elution with 3x FLAG peptide (150 mM NaCl, 0.05% Tween, 150 ng/μl FLAG peptide (Sigma-Aldrich)). Eluates were boiled with Laemmli buffer and fractionated by SDS-PAGE.

### FRAP

*53BP1*^*-/-*^ MCF-7 cells were stably transduced with an mClover2-tagged fragment of 53BP1 encompassing amino acids 1107–1711 of the wild type protein. Two days before FRAP, 2.5 × 10^5^ cells were seeded on a 35 mm glass-bottom plate. Cells were irradiated (5 Gy) with an X-ray source to induce IRIF formation 2 h before imaging. Individual foci were selected and 10 prebleach images were acquired prior to a single bleach pulse with an Argon laser at a laser transmission of 100%. Immediately after bleaching, 120 images were acquired in 5 s intervals at 0.5% laser intensity. All acquisition files were processed in ImageJ with the Stackreg plugin to account for nuclear migration in the recovery period. Regions of interest encompassing the bleached area, the entire nuclei and a section of background were selected and mean intensity values were quantified for each in ImageJ. These values were then normalized and fitted using the easyFRAP software^52^. All acquisitions were performed on a Leica SP8 SMD X confocal microscope.

Initial rate values were calculated based on curve of best fit generated in MATLAB according to the following equation.

$$F\left( x \right) = a ^\ast {\mathrm{exp}}\left( {b ^\ast x} \right) + c ^\ast {\mathrm{exp}}\left( {d ^\ast x} \right)$$

Curves of best fit were generated with the following coefficient values (with 95% confidence bounds), mC2-f53BP1: *a* = 0.3191 (0.3083, 0.33), *b* = 0.0006296 (0.0005654, 0.0006938), *c* = −0.3105 (−0.32, −0.301), *d* *=* −0.009148 (−0.009856, −0.00844), goodness of fit, SSE = 0.00501, R-square = 0.9964, adjusted R-square = 0.9963, RMSE = 0.006572; mC2-f53BP1^ODm^: *a* = 0.4695 (0.463, 0.4759), *b* = 0.000618 (0.0005858, 0.0006501), *c* = −0.5312 (−0.5555, −0.5069), *d* = −0.02533 (−0.02711, −0.02355), goodness of fit, SSE = 0.01578, R-square = 0.9909, adjusted R-square = 0.9906, RMSE = 0.01167; mC2-f53BP1^LC8m^: *a* = 0.4598 (0.4451, 0.4745), *b* = 0.0004644 (0.0004064, 0.0005225), *c* = −0.4654 (−0.4778, −0.4531), *d* = −0.008194 (−0.008687, −0.007701), SSE = 0.00528, R-square = 0.9979, adjusted R-square = 0.9979, RMSE = 0.006747. Rates for x = 10 s are presented in Fig. 3e.

### Expression and purification of 53BP1

Smt3-His_6_-tagged fragments (amino acids 1131–1292) of wild type (LC8-OD), OD-mutated (ODm-LC8), LC8-mutated (OD-LC8m), double mutant (LC8m-ODm) 53BP1, and DYNLL1 were expressed from pET-17b in *E. coli* BL21(DE3). Cells were lysed by sonication in 20 mM HEPES pH 7.5, 250 mM NaCl, and 0.25 mM TCEP. Lysates were clarified by centrifugation and the supernatant was applied to a TALON IMAC column (Supplementary Fig. 2a) or Ni-NTA agarose beads (Fig. 3f). Bound complexes were washed with 20 mM HEPES pH 7.5, 250 mM NaCl, 0.25 mM TCEP, supplemented with 10 mM imidazole and then eluted by increasing the imidazole concentration to 250 mM. Equal concentrations of eluate were then fractionated over a Superdex S200 column (Supplementary Fig. 2a).

### In vivo tumourigenesis studies

All animal experiments were approved by the Animal Ethics Committee of The Netherlands Cancer Institute (Amsterdam, the Netherlands) and performed in accordance with the Dutch Act on Animal Experimentation (November 2014). KB1P4 tumour organoids were established previously and cultured in AdDMEM/F12 supplemented with 1 M HEPES (Sigma), GlutaMAX (Invitrogen), penicillin/streptomycin (Gibco), B27 (Gibco), 125 μM N-acetyl-L-cysteine (Sigma), 50 ng/mL murine epidermal growth factor (Invitrogen) (Duarte et al.^43^). Tumour organoid transduction was performed by spinoculation with pLentiCRISPRv2 lentiviral constructs in which either no gRNA or a gRNA targeting ASCIZ was cloned. Following puromycin selection, modified organoids were collected, incubated with TripLE at 37 °C for 5ʹ, dissociated into single cells, washed in PBS, resuspended in tumour organoid medium and mixed in a 1:1 ratio of tumour organoid suspension and BME in a cell concentration of 10^4^ cells per 40 μl. Subsequently, 10^4^ cells were transplanted in the fourth right mammary fat pad of 6–9-week-old female NMRI nude mice. Mammary tumour size was measured by caliper measurements and tumour volume was calculated (0.5  × length × width^2^). Treatment of tumour bearing mice was initiated when tumours reached a size of 50–100 mm^3^, at which point mice were stratified into the untreated (*n* = 8) or olaparib treatment group (*n* = 8). Olaparib was administered at 100 mg/kg intraperitoneally for 80 consecutive days. Animals were sacrificed with CO_2_ when the tumour reached a volume of 1500 mm^3^. Sample sizes were not pre-determined and no blinding or randomization was employed during analysis.

### Quantification and statistical analysis

Prism 6 (GraphPad Software Inc.) was used for statistical analysis and production of all graphs and dot plots. The relevant statistical methods and measures of significance for each experiment are detailed in the figure legends. Cell Profiler (Broad Institute) was used for automated quantitation of immunofluorescence data. Normalization of FRAP recovery curves was performed using the easyFRAP software and curves of best fit were generated in MATLAB.

## Supplementary information

Supplementary Information

Source Data

## Data Availability

The source data underlying Figs. 1d–f, 2b and d, 3b–d and f, 4d, f and g, 5a and b, and Supplementary Figures 1c and d, 2b–e, 3a–d, 4b–f, 5c and e, and 6a and c are provided as a Source Data file. All other original data and code that supports the findings of this study are available from the corresponding author upon reasonable request.
