# Supplementary material for: The ASCIZ-DYNLL1 axis promotes 53BP1-dependent non-homologous end joining and PARP inhibitor sensitivity
Source: Nat Commun. 2018 Dec 17;9:5406. doi: 10.1038/s41467-018-07855-x (PMC6297349; doi:10.1038/s41467-018-07855-x)
Supplement: Supplementary file 1 — Supplementary Information [file 41467_2018_7855_MOESM1_ESM.pdf]

SUPPLEMENTARY INFORMATION

The ASCIZ-DYNLL1 axis promotes 53BP1-dependent NHEJ and PARP inhibitor sensitivity

Jordan R. Becker, Raquel Cuella-Martin, Marco Barazas, Rui Liu, Catarina Oliveira, Antony W. Oliver, Kirstin Bilham, Abbey B. Holt, Andrew N. Blackford, Jörg Heierhorst, Jos Jonkers, Sven Rottenberg, J. Ross Chapman

Contents:

Supplementary Figure 1 (Related to Figure 1): Screen of N-terminal 53BP1 truncations for IRIF formation.

Supplementary Figure 2 (Related to Figure 1): LC8 motif mutation does not intrinsically impair 53BP1 oligomerization or RIF1 foci formation.

Supplementary Figure 3 (Related to Figure 1): DYNLL1 regulation of 53BP1 is cell cycle-independent.

Supplementary Figure 4 (Related to Figure 2): DYNLL1 protein is undetectable in *ΔDynll1* B cells.

Supplementary Figure 5 (Related to Figure 4): *DYNLL1* and *ASCIZ* gene-editing provides a growth advantage in olaparib treated *Brca1*$^{-/-}$ *p53*$^{-/-}$ tumour cells.

Supplementary Figure 6 (Related to Figure 4): DYNLL1 expression sensitizes *ASCIZ*-edited cells to olaparib.

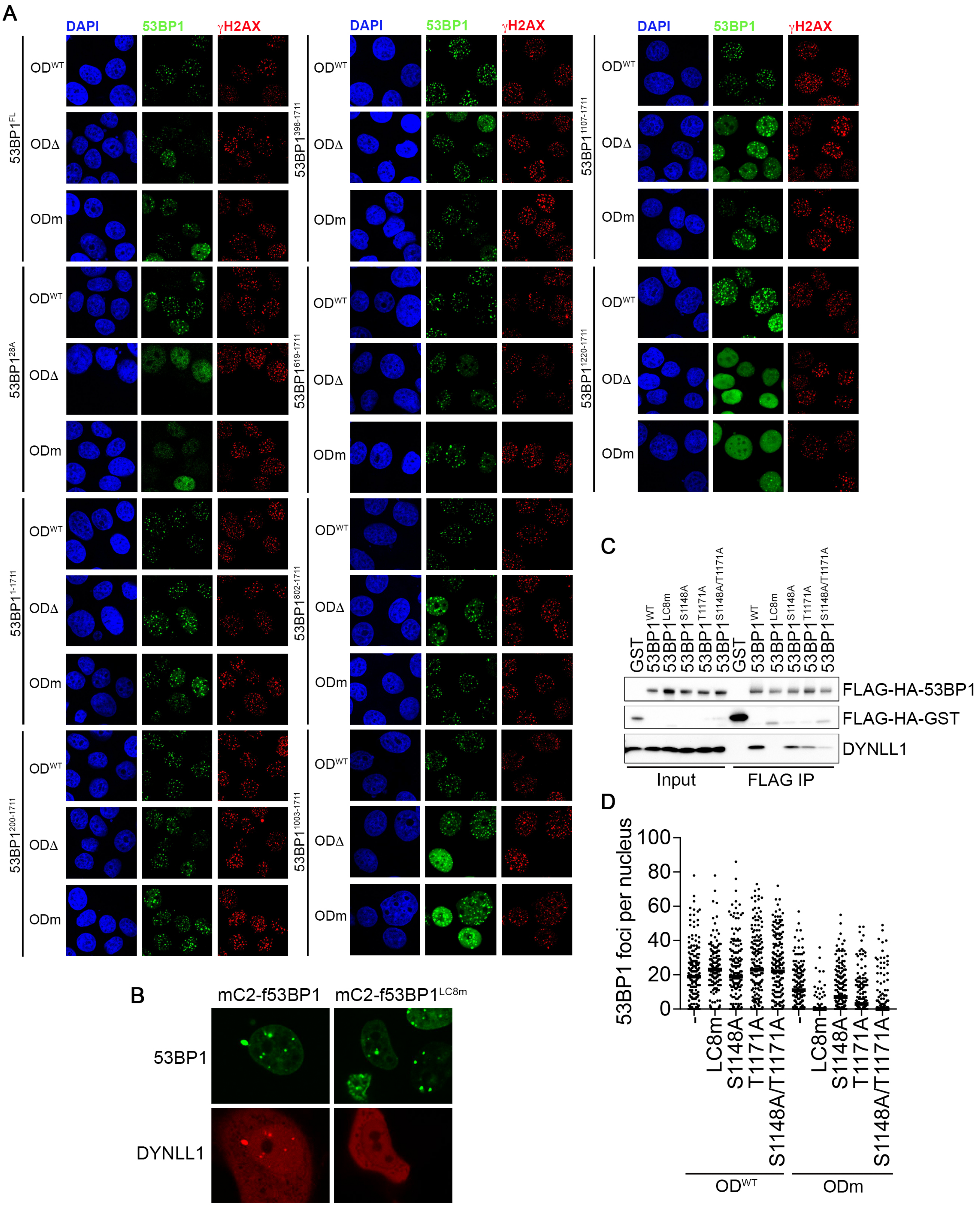

Supplementary Figure 1

Supplementary Figure 1 (Related to Figure 1): Screen of N-terminal 53BP1 truncations for IRIF formation.

(A) *53BP1*$^{-/-}$ MCF-7 cells were transduced with lentivirus encoding the indicated FLAG-HA-53BP1 alleles. The resulting cells were seeded on glass coverslips 48h before irradiation (5 Gy). Cells were fixed 4h after irradiation and stained with anti-HA and anti-γH2AX antibodies. Images were acquired on a Zeiss LSM510 META confocal imaging system.

(B) *53BP1*$^{-/-}$ MCF-7 cells were stably transduced with mC2-f53BP1 (wild type) or mC2-f53BP1^{LC8m}. Cells were then transiently transfected with DsRed-DYNLL1 24 h before live imaging. Data, representative of *n=2* independent experiments.

(C) FLAG-HA-53BP1 immuno-complexes were isolated from whole cell extracts prepared from HEK 293T cultures, 48 h following transfection with indicated control (GST) or 53BP1 expression plasmids. Representative data, *n=3* independent experiments.

(D) Quantification of 53BP1 foci number in MCF7 *53bp1*$^{-/-}$ cells complemented with the indicated transgenes 4 h after 5 Gy irradiation. ≥139 nuclei were scored per condition. Black bar represents median value.

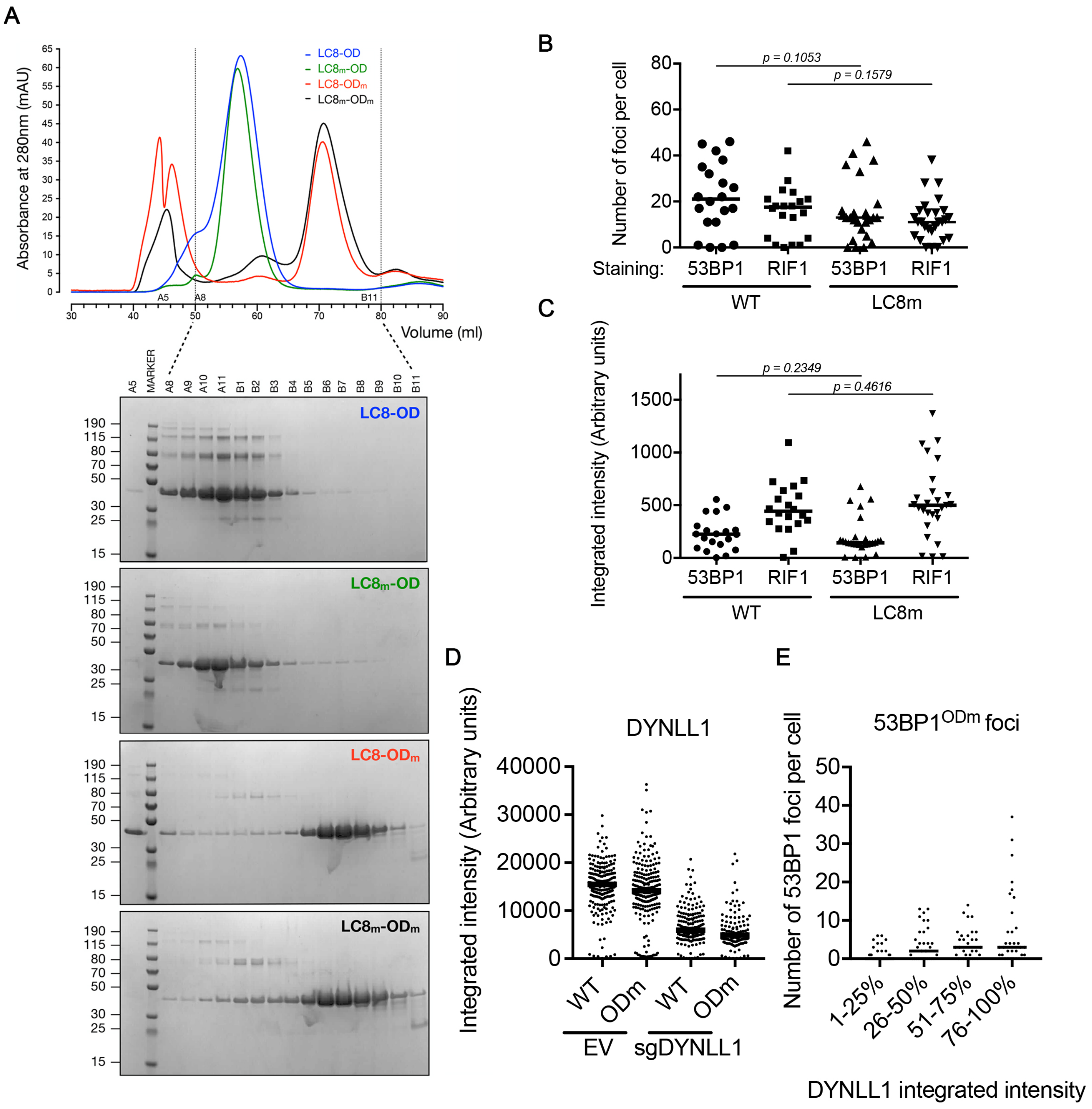

Supplementary Figure 2

Supplementary Figure 2 (Related to Figure 1): LC8 motif mutation does not intrinsically impair 53BP1 oligomerization or RIF1 foci formation.

(A) 53BP1 fragments encompassing the LC8 motifs and OD (a.a. 1131-1292) with C-terminal Smt3-His_6 tags were expressed in *E. coli* and purified using a TALON IMAC column. Wild type (LC8-OD), OD-mutated (ODm-LC8), LC8-mutated (OD-LC8m), or double mutant (LC8m-ODm) fragments were prepared in this manner and fractionated over a Superdex S200 column.

(B) MCF-7 cells expressing either wild type 53BP1 or $53BP1^{LC8m}$ were irradiated (5 Gy) 4h prior to fixation and staining. Number of RIF1 and 53BP1 foci per cell in MCF-7 cells expressing either wild type 53BP1 or $53BP1^{LC8m}$ as indicated. Significance was determined by Mann-Whitney U Test.

(C) Relative intensity of RIF1 and 53BP1 foci (arbitrary units). Foci number and intensity were quantified using Cell Profiler. Significance was determined by Mann-Whitney U Test.

(D) Per nucleus integrated intensity of DYNLL1 signal. Related to Figure 1E. Black bars represent median values.

(E) MCF-7 cells expressing FLAG-HA-$53BP1^{ODm}$ transduced with a CRISPR/Cas9 lentivirus co-expressing *DYNLL1*-specific sgRNA. Cultures were irradiated (5 Gy) 72h after infection and fixed 4h after irradiation. Fixed cells were probed with anti-HA and anti-DYNLL1 antibodies. Foci number and intensity were measured using CellProfiler. DYNLL1 signal intensity is binned into quartiles and sorted from lowest to highest intensity. Positive correlation between foci number and intensity was determined by ordinary 1-way ANOVA test for trend ($p < 0.0001$).

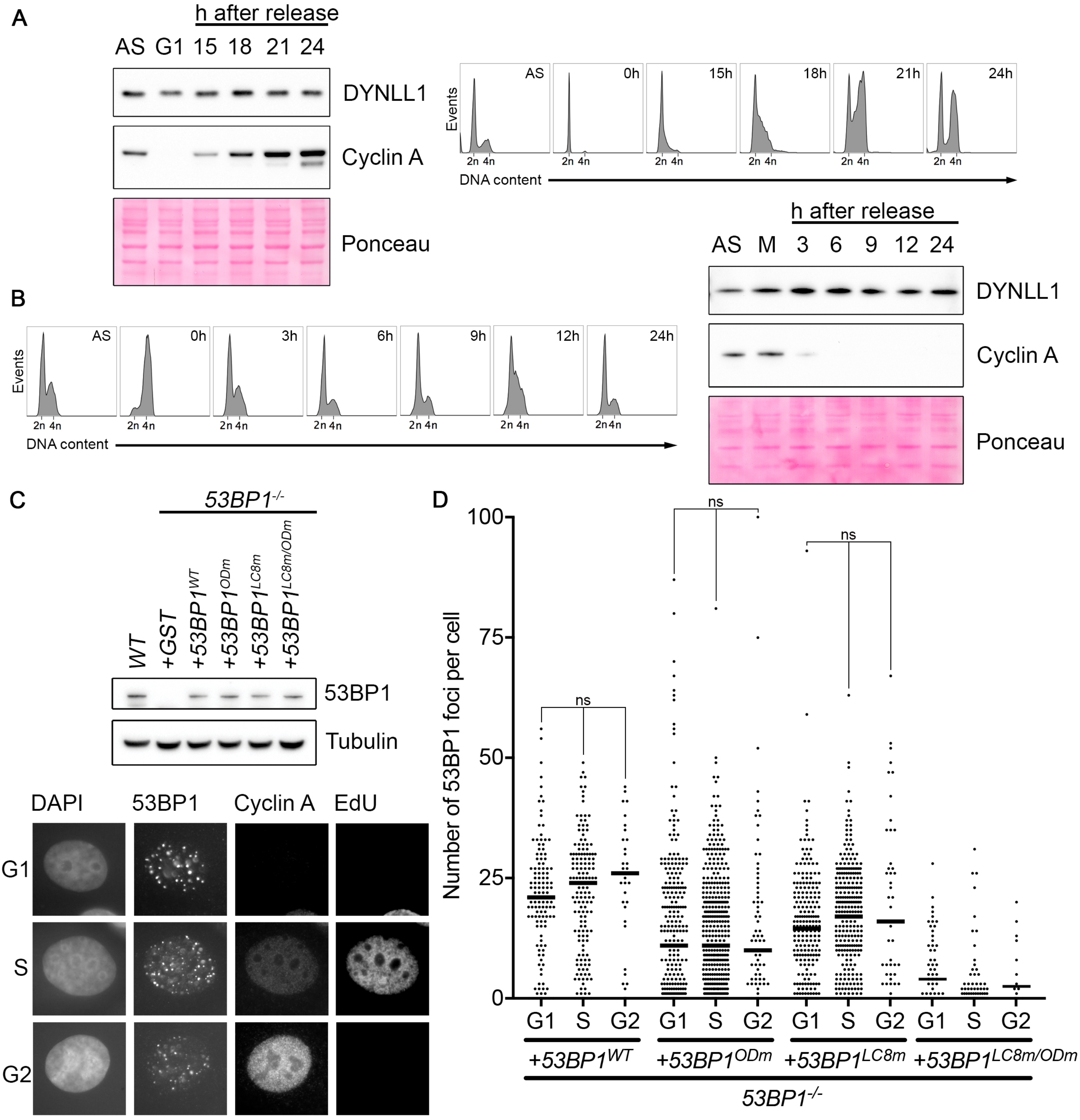

Supplementary Figure 3

Supplementary Figure 3 (Related to Figure 1): DYNLL1 regulation of 53BP1 is cell cycle-independent

(A) RPE1 cells were released into serum containing medium from a G0 serum-arrest block (>95% G0 purity). Samples taken at the indicated time points were immunoblotted with indicated antibodies. DNA content was measured by propidium iodide staining and flow cytometry (right).

(B) RPE1 cells synchronized in G2/M with nocodazole and mitotic shake-off, were released into mitosis, and protein samples collected at the indicated time points following nocodozole removal. DNA content was measured by propidium iodide staining and flow cytometry (left).

(C) Western blot analysis of the cell lines utilized in (D). Representative images of G1, S and G2 cells as discriminated by Cyclin A and EdU staining (below).

(D) Quantification of 53BP1 foci number 4 h after 5 Gy irradiation. Significance was determined by Mann-Whitney U Test (*ns, p>0.05*).

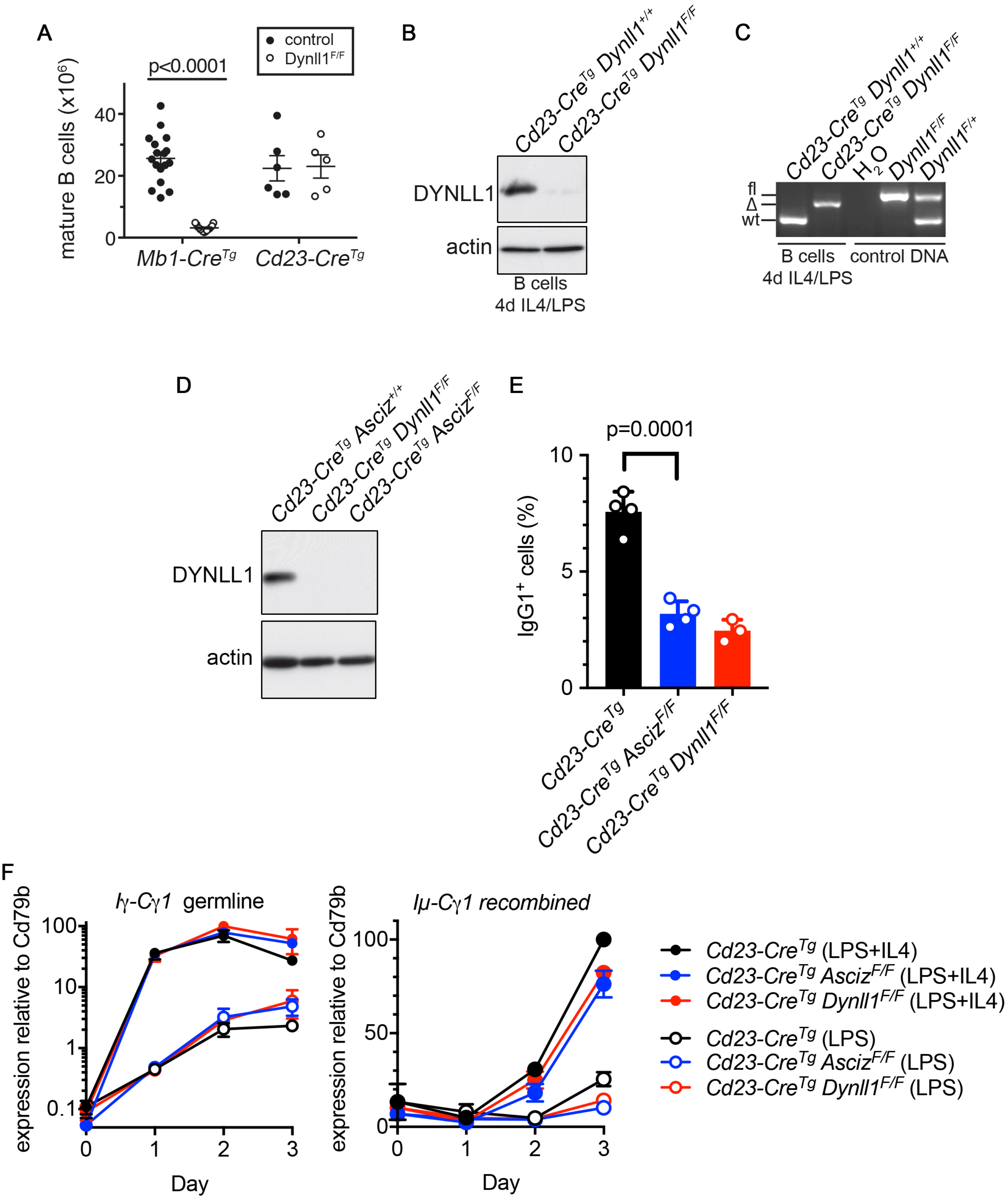

Supplementary Figure 4

Supplementary Figure 4 (Related to Figure 2): DYNLL1 protein is undetectable in *ΔDynll1* B cells.

(A) Quantification of mature B cell numbers in *Mb1-Cre*^{Tg}, *Mb1-Cre*^{Tg} Dynll1$^{F/F}$, *Cd23-Cre*^{Tg}, and *Cd23-Cre*^{Tg} *Dynll1*$^{F/F}$. Significance was determined by Mann-Whitney U Test.

(B) Lysates prepared from *Cd23-Cre*^{Tg} *Dynll1*$^{+/+}$ control and *Cd23-Cre*^{Tg} *Dynll1*$^{F/F}$ -deleted B cells were fractionated by SDS-PAGE and probed with anti-actin and anti-DYNLL1 antibodies as indicated.

(C) PCR validation of complete *Dynll1*$^{F/F}$ recombination in mature *Cd23-Cre*^{Tg} *Dynll1*$^{F/F}$ B splenocytes.

(D) Lysates prepared from *Cd23-Cre*^{Tg} *Asciz*$^{+/+}$ control, *Cd23-Cre*^{Tg} *Asciz*$^{F/F}$*-deleted*, and *Cd23-Cre*^{Tg} *Dynll1*$^{F/F}$*-deleted* B cells were fractionated by SDS-PAGE and probed with anti-actin and anti-DYNLL1 antibodies as indicated.

(E) *In vitro* CSR to IgG1 in *Cd23-Cre*^{Tg} *Asciz*$^{F/F}$*, Cd23-Cre*^{Tg} *Dynll1*$^{F/F}$ and control *Cd23-Cre*^{Tg} mature B splenocytes, 96 h following stimulation with LPS and IL-4. Data, *n=4* mice. Significance was determined by unpaired two-tailed t test.

(F) Relative expression of germline (left) and recombined (right) transcripts in purified B cells from *Cd23-Cre*^{Tg}*, Cd23-Cre*^{Tg} *Asciz*$^{F/F}$*,* or *Cd23-Cre*^{Tg} *Dynll1*$^{F/F}$ mice cultured with LPS (15 μg/mL) and IL-4 (1/100) for 0 to 3 days. Normalised to Cd79b loading control and expressed as a percentage of maximum signal. Data, *n=2* mice per condition, mean ± range.

A

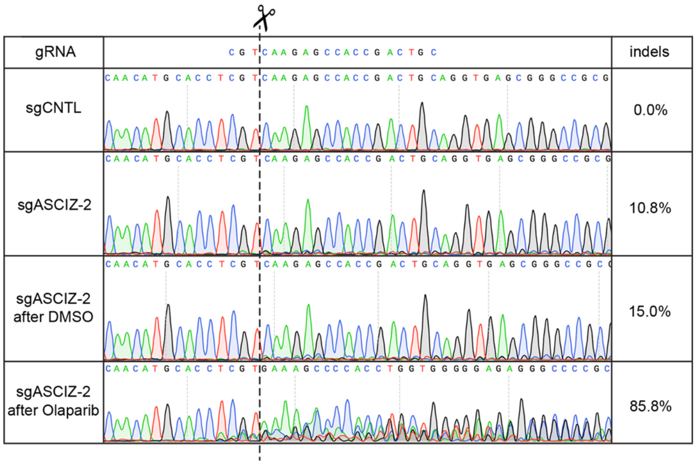

B

KB1P-G3 Trial 1

	naïve (% of alleles with indels)	DMSO (change in % indel frequency)	75 nM Olaparib (change in % indel frequency)
sgASCIZ-1	22.7	-17.4	45.2
sgASCIZ-2	10.8	4.2	75.0
sgDYNLL1-1	23.6	-6.6	47.3
sgDYNLL1-2	25.9	-7.8	51.1

KB1P-G3 Trial 2

	naïve (% edited alleles)	DMSO (change in % indel frequency)	75 nM Olaparib (change in % indel frequency)
sgASCIZ-1	68.1	-8.9	21.7
sgASCIZ-2	72.6	-6.4	15.6
sgDYNLL1-1	83.7	-30.7	8.4
sgDYNLL1-2	88.1	-12.7	0.5

KB1P-G3 Trial 3

	naïve (% edited alleles)	DMSO (change in % indel frequency)	75 nM Olaparib (change in % indel frequency)
sgASCIZ-1	4.9	16.7	58.6
sgASCIZ-2	5.0	27.3	38.0
sgDYNLL1-1	14.7	17.3	30.9
sgDYNLL1-2	12.3	13.8	42.6

C

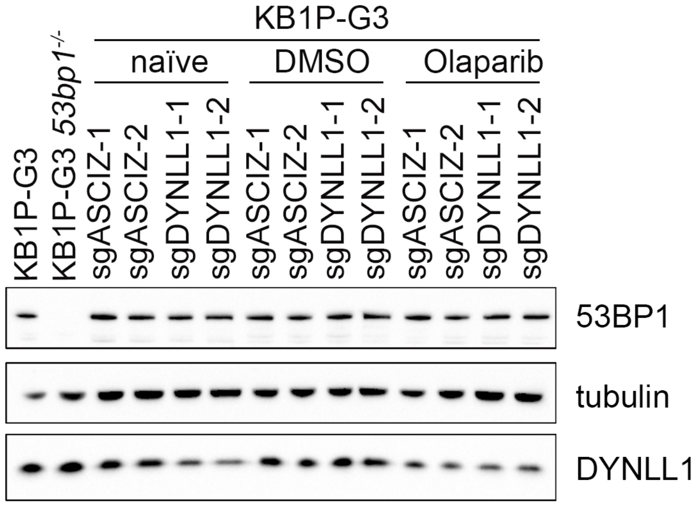

D

KB1P-G3 *53bp1*$^{-/-}$ Trial 1

	naïve (% edited alleles)	DMSO (change in % indel frequency)	75 nM Olaparib (change in % indel frequency)
sgASCIZ-1	2.3	7.8	5.2
sgASCIZ-2	2.2	5.3	5.9
sgDYNLL1-1	8.7	5.8	3.1
sgDYNLL1-2	6.9	6.5	5.4

KB1P-G3 *53bp1*$^{-/-}$ Trial 2

	naïve (% edited alleles)	DMSO (change in % indel frequency)	75 nM Olaparib (change in % indel frequency)
sgASCIZ-1	46.0	-0.2	1.6
sgASCIZ-2	51.4	-6.2	0.9
sgDYNLL1-1	69.9	-25.5	-5.3
sgDYNLL1-2	75.5	-11.9	-28.6

E

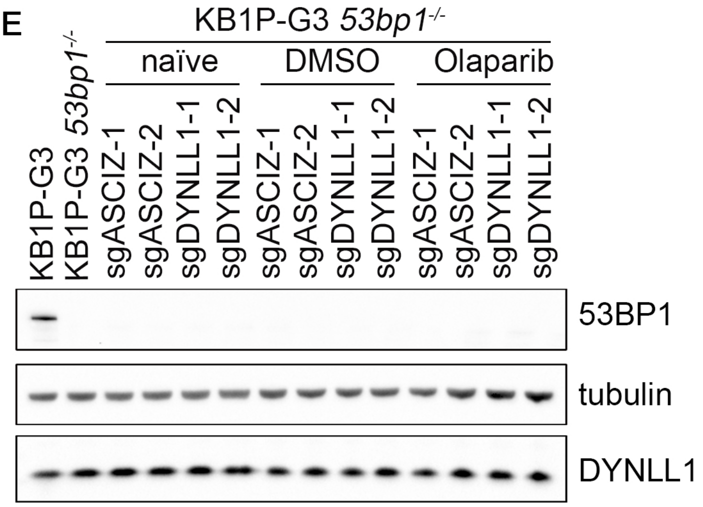

F

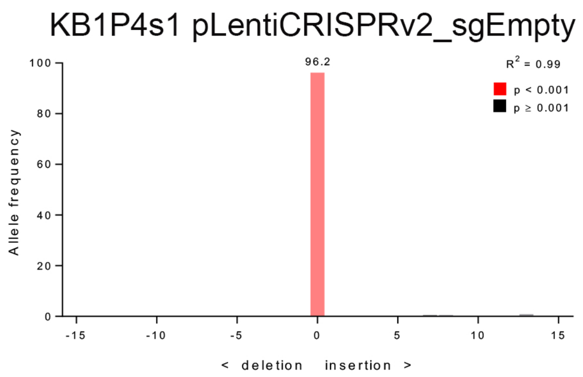

G

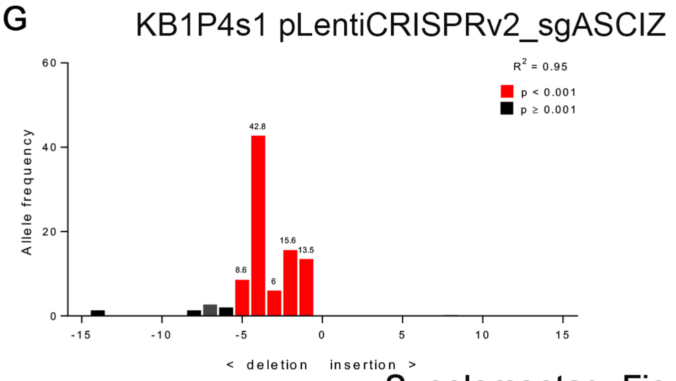

Supplementary Figure 5

Supplementary Figure 5 (Related to Figure 4): *DYNLL1* and *ASCIZ* gene-editing provides a growth advantage in olaparib treated *Brca1*$^{-/-}$ *p53*$^{-/-}$ tumour cells.

(A) Representative Sanger sequencing chromatograms generated from cell populations transduced with sgCNTL or sgASCIZ encoding virus and after outgrowth in DMSO or olaparib as indicated.

(B) Additional independent trials of the experiment presented in Figure 4B. Percentage of edited alleles after transduction with the indicated gRNAs and antibiotic selection (naïve), after 8 days outgrowth in DMSO (negative control), or after 10 days outgrowth in the presence of olaparib (75 nM).

(C) Whole cell extracts were isolated from KB1P-G3 cells after transduction with the indicated gRNAs and antibiotic selection (naïve), after 8 days outgrowth in DMSO (negative control), or after 10 days outgrowth in the presence of olaparib (75 nM). Extracts were fractionated by SDS-PAGE and probed with antibodies specific to the indicated antigens.

(D) Additional independent trials of the experiment presented in Figure 4E. Percentage of edited alleles after transduction with the indicated gRNAs and antibiotic selection (naïve), after 8 days outgrowth in DMSO (negative control), or after 10 days outgrowth in the presence of olaparib (75 nM) is presented for 2 independent experiments (trials 2 and 3)

(E) Whole cell extracts were isolated from KB1P-G3 *53bp1*$^{-/-}$ cells after transduction with the indicated gRNAs and antibiotic selection (naïve), after 8 days outgrowth in DMSO (negative control), or after 10 days outgrowth in the presence of olaparib (75 nM). Extracts were fractionated by SDS-PAGE and probed with antibodies specific to the indicated antigens.

(F) and (G) TIDE analysis histograms of control (C) and ASCIZ-edited (D) organoids used for transplantation in Figure 4F-G.

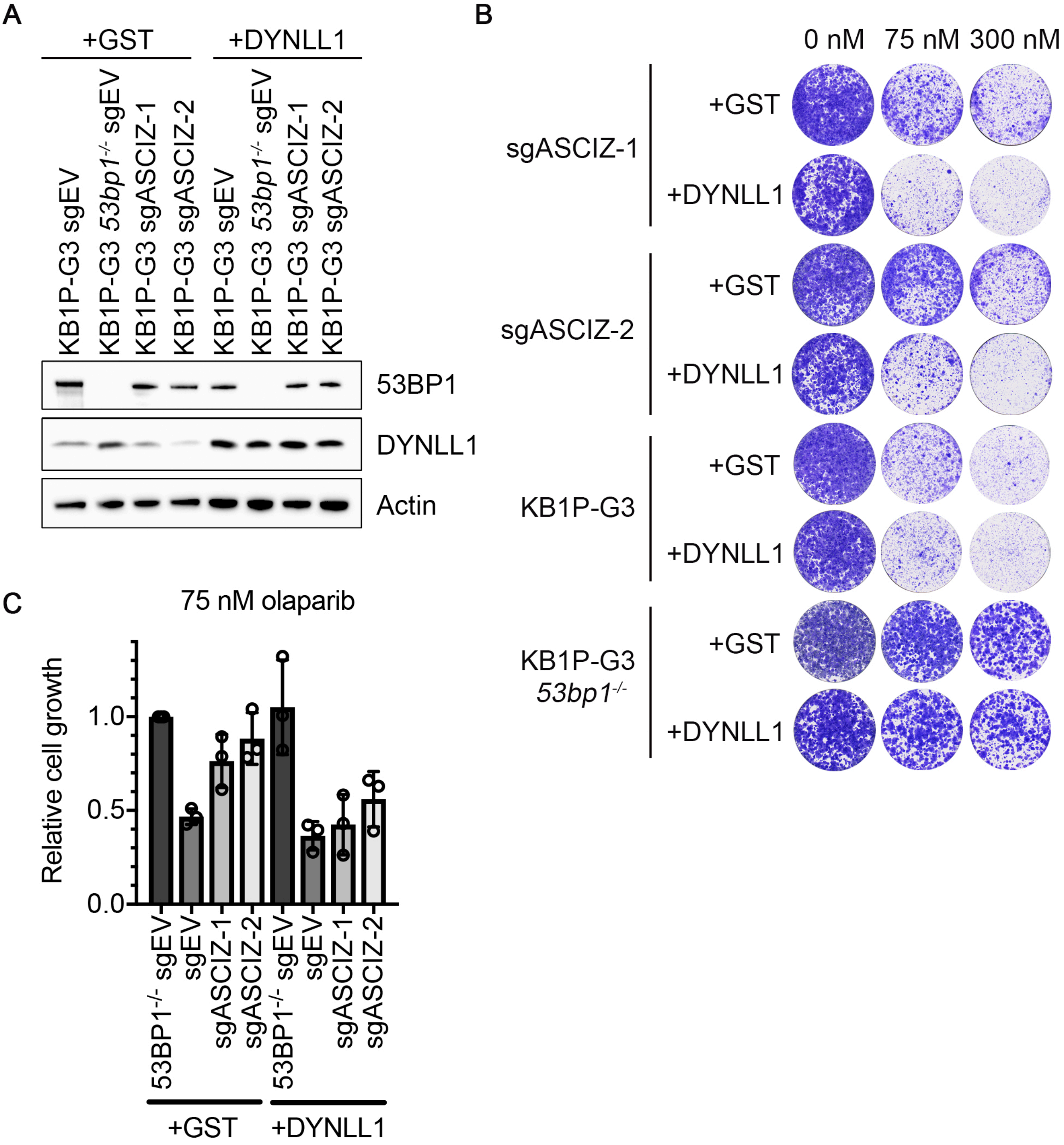

Supplementary Figure 6

Supplementary Figure 6 (Related to Figure 4). DYNLL1 expression sensitizes *Asciz*-edited cells to olaparib.

(A) Whole cell extracts of control and *Asciz*-edited KB1P-G3 cells were transduced with GST (control) or DYNLL1. Extracts were fractionated by SDS-PAGE and western blotted with the indicated antibodies.

(B) Representative images of growth in either DMSO or olaparib.

(C) Quantification of growth in olaparib (75 nM) after transduction with DYNLL1. Quantification of *n=3* independent experiments, each with 3 technical replicates. Mean ± SD.
